# Adducin‐1 Facilitates Influenza Virus Endosomal Trafficking and Uncoating by Regulating Branched Actin Dynamics and Myosin IIB Activity

**DOI:** 10.1002/advs.202417318

**Published:** 2025-06-05

**Authors:** Meijun Jiang, Jiahui Zou, Yaoming Jin, Chenjun Jiang, Shaoyu Tu, Tong Chen, Jinli Guo, Yanqing Cheng, Meilin Jin, Huanchun Chen, Hongbo Zhou

**Affiliations:** ^1^ National Key Laboratory of Agricultural Microbiology College of Veterinary Medicine Huazhong Agricultural University Wuhan Hubei 430070 P. R. China; ^2^ Frontiers Science Center for Animal Breeding and Sustainable Production Wuhan Hubei 430070 P. R. China; ^3^ Hubei Hongshan Laboratory Wuhan Hubei 430070 P. R. China; ^4^ Key Laboratory of Preventive Veterinary Medicine in Hubei Province The Cooperative Innovation Center for Sustainable Pig Production Wuhan Hubei 430070 P. R. China

**Keywords:** ADD1, Arp2/3, endosomal trafficking, F‐actin, Influenza virus, uncoating

## Abstract

Actin‐ and microtubule‐based transport systems are essential for the trafficking of endocytic viruses and cargoes. Microtubules facilitate long‐distance transport; however, the precise role of actin dynamics and its regulators during virus entry, particularly in the transit process, remains elusive. Here, Adducin‐1 (ADD1) is identified as a key regulator of actin dynamics, as demonstrated by real‐time monitoring of quantum dot (QD)‐labeled influenza A virus (IAV) movement. ADD1 deletion increases actin density around endocytic vesicles, disrupting general vesicular trafficking and inhibiting the replication of diverse endocytic viruses. Mechanistically, endocytic viruses or cargoes trigger the phosphorylation of ADD1 at Ser726, which reduces the density of actin branches for effective transport. Additionally, the physical force required for IAV capsid dissociation is influenced by ADD1. Collectively, the study identifies a basic actin dynamics event with broad relevance to endocytic viruses or cargo trafficking and represents ADD1 as a potential target for developing broad‐spectrum antiviral strategies.

## Graphical abstract

In this study, a novel mechanism is unveiled by which ADD1, acting as a molecular switch, coordinates actin branch dynamics and the transport of endocytic viruses and cargoes. Phosphorylation of ADD1 at Ser726 reduces actin branch density, enhancing endosome fusion and attachment to microtubules. EG‐011 treatment and ADD1 knockdown reduced influenza virus replication in vivo and in vitro.

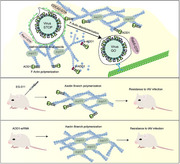

## 1 Introduction

The majority of enveloped viruses are significant human pathogens, with influenza A virus (IAV) and SARS‐CoV‐2 causing pandemics that result in millions of deaths.^[^ ^1^, ^2^ ^]^ To achieve effective infection and replication, enveloped viruses generally utilize endocytosis for uptake and are transported toward the perinuclear region via intracellular vesicles. This process heavily relies on the dynamic reorganization of cytoskeletal elements. Disruption of actin nucleators or F‐actin polymerization results in the enlargement and elongation of endosomes and impedes the transport of the virus from early to late endosome,^[^ ^3^, ^4^, ^5^ ^]^ suggesting a critical link between virus trafficking and actin dynamics assembly. Thus, a comprehensive understanding of how actin dynamics assembly is orchestrated during enveloped virus entry is imperative for the development of effective broad‐spectrum antiviral targets.

Upon IAV attachment to the host cell via the viral glycoprotein haemagglutinin (HA), the coordinated activation of signaling pathways would be triggered to regulate the dynamic assembly of F‐actin.^[^ ^6^, ^7^, ^8^ ^]^ The subsequent internalization of IAV mainly depends on clathrin‐mediated endocytosis (CME), where nucleation‐promoting factors (NPFs) are recruited to the viral endocytosis sites and initiate the assembly of branched actin networks mediated by the actin‐related protein 2/3 (Arp2/3) complex, thereby facilitating the plasma membrane invagination, the invaginated pit elongation, and the nascent vesicle and plasma membrane constriction.^[^ ^9^, ^10^, ^11^, ^12^, ^13^, ^14^ ^]^ The vesicle is then moved away from the membrane and transported along myosin‐driven microfilaments and dynein‐driven microtubules.^[^ ^15^ ^]^ The progressively decreasing pH within the vesicle triggers a conformational change of viral HA, promoting the fusion of the viral envelope with the endosomal membrane and exposing the viral core and unanchored ubiquitin (Ubs) chains into the cytosol.^[^ ^16^, ^17^ ^]^ Subsequently, HDAC6, acting as a linking molecule, binds to the capsid‐associated Ubs and cytoskeletal motors (myosin IIB and dynein), generating physical forces to dissociate viral capsid.^[^ ^18^ ^]^ Viral ribonucleoproteins (vRNPs) are then released and imported into the nucleus for genome transcription and replication. Collectively, enveloped virus entry is intricately linked with actin dynamics. However, the role of actin dynamics following vesicle‐packaged virus formation remains unclear, and the key regulatory factors involved in this process have yet to be identified.

As an actin‐capping protein, Adducin‐1 (ADD1) was identified as a crucial host factor for IAV replication through our previous genome‐wide CRISPR‐Cas9 screening,^[^ ^19^ ^]^ ranking fourth in the screen and potentially serving as a pivotal regulator of actin dynamics in viral trafficking and replication. ADD1 regulates actin assembly dynamics by capping the fast‐growing barbed ends of actin filaments, bundling these filaments, and recruiting spectrin to form spectrin‐actin networks.^[^ ^20^ ^]^ The stability of the spectrin‐actin network prevents actin filament depolymerization.^[^ ^21^, ^22^, ^23^ ^]^ Phosphorylation at Ser726 in the C‐terminal tail domain of ADD1 disrupts its actin‐capping activity, causing the dissociation of the spectrin‐actin network. The disassembly of this structure exposes actin filaments, enabling actin‐depolymerizing proteins (e.g., cofilin) to bind to and sever actin filaments.^[^ ^20^, ^21^, ^23^, ^24^, ^25^, ^26^, ^27^ ^]^ This process accelerates actin filament depolymerization and promotes actin cytoskeletal remodeling. However, whether ADD1 coordinates endocytic trafficking and F‐actin dynamics has never been investigated.

In this study, we identified that ADD1 is involved in the dynamic reorganization of F‐actin following vesicle‐packaged virus formation. Virus‐induced dynamic rearrangement of F‐actin exhibits a tendency to undergo initial polymerization followed by depolymerization. The branched actin at sites of endocytosis promotes the formation of vesicle‐packaged virus and subsequently acts as a physical barrier to inhibit viral transport. Endocytic viruses, including IAV, vesicular stomatitis virus (VSV), and porcine epidemic diarrhea virus (PEDV) or cargoes like EGF could elevate the phosphorylation of ADD1 at Ser726, which reduces the density of actin branches around endocytic vesicles, facilitating the fusion of virus‐containing vesicles with early endosomes and their attachment to microtubules for rapid movement. Meanwhile, ADD1 facilitates IAV uncoating by modulating the RhoA/MLC/myosin IIB axis. In conclusion, our study unveils ADD1‐mediated actin branch dynamics with broad relevance to the transport of endocytic viruses or cargoes and identifies ADD1 as an undescribed target for the development of broad‐spectrum antiviral strategies.

## 2 Results

### 2.1 ADD1 Promotes the Replication of Different Host‐Origin IAV Strains

**Figure 1:**
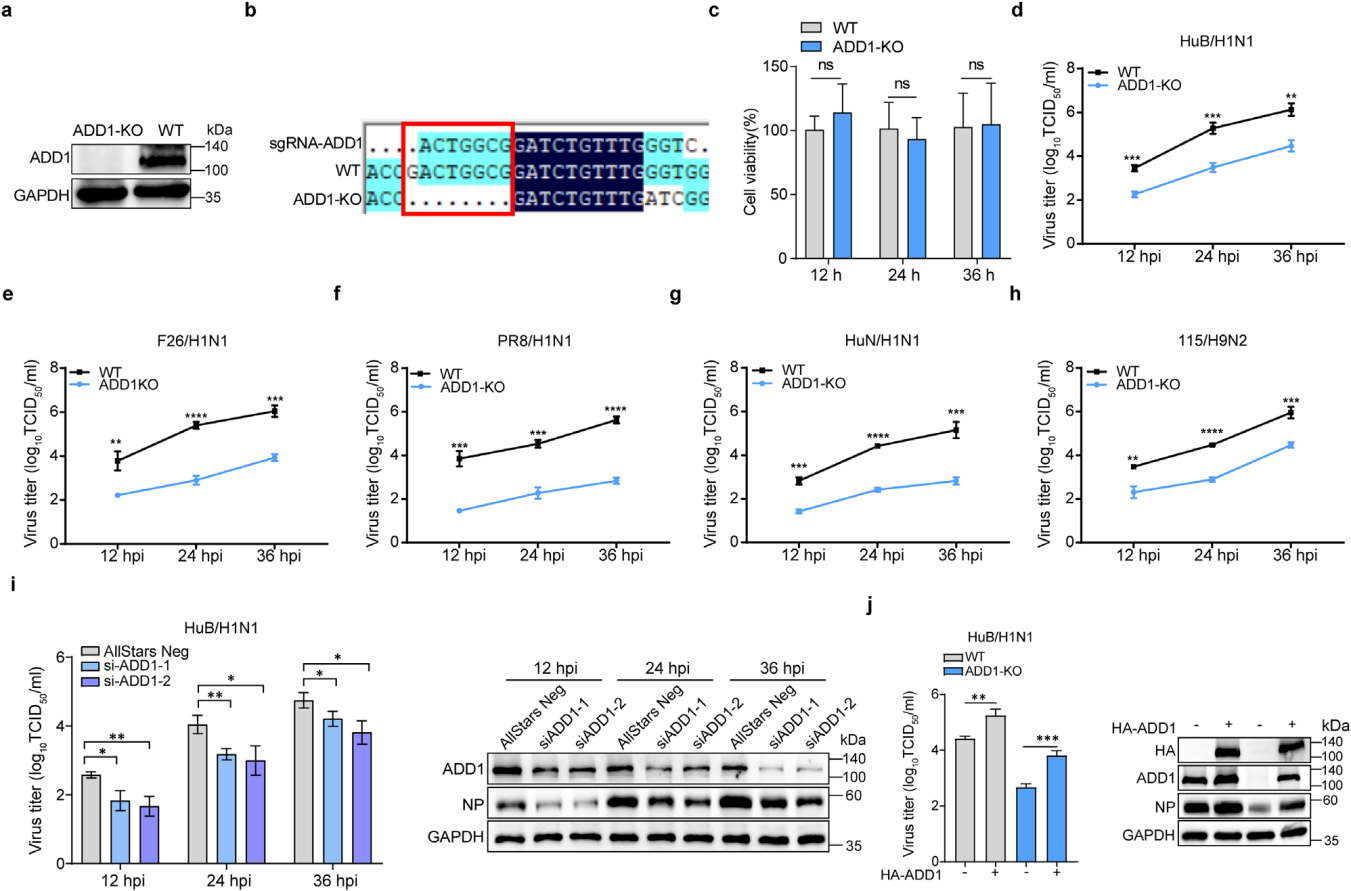
ADD1 promotes the replication of multiple influenza virus strains. a,b) The knockout efficiency of ADD1 in PK‐15 cells was measured by Western blot assay (a) and Sanger sequencing (b) (*n* = 3). c) Cell viability of WT and ADD1‐KO PK‐15 cells was determined by CCK‐8 detection (*n* = 8). d–h) WT and ADD1‐KO PK‐15 cells were respectively infected with HuB/H1N1 (MOI = 0.01) (d), F26/H1N1 (MOI = 0.01) (e), PR8/H1N1 (MOI = 0.01) (f), HuN/H1N1 (MOI = 0.01) (g), or 115/H9N2 (MOI = 0.01) (h). The infected cell supernatants were subsequently harvested at the indicated times, and virus titers were determined by TCID_50_ assay (*n* = 3). i) PK‐15 cells were treated with AllStars Negative Control siRNA or siADD1, followed by infection with HuB/H1N1 (MOI = 0.01), and virus titers were determined at the indicated times. Immunoblotting was performed to assess the expression levels of ADD1 and NP protein, with grayscale analysis for quantification (*n* = 3). j) WT and ADD1‐KO PK‐15 cells were transfected with HA vector or HA‐ADD1 for 24 h, followed by infection with HuB/H1N1 (MOI = 0.01) to assess viral titers. Immunoblotting was performed to assess the expression levels of HA, ADD1 and NP protein, with grayscale analysis for quantification (*n* = 3). Data are presented as mean ± Standard Deviation (SD). Statistical analysis was performed using unpaired, two‐tailed Student's t‐test. *P < 0.05; **P < 0.01; ***P < 0.001; ****P < 0.0001; *ns*, not significant.

To elucidate the role of ADD1 in IAV infection, we generated ADD1 knockout (ADD1‐KO) porcine kidney‐15 (PK‐15) cells using the CRISPR/Cas9 system, and ADD1 knockout had no major effect on cell viability (**Figure** **1**a–c). Subsequently, the effect of ADD1 on IAV infection was evaluated with A/swine/Hubei/221/2016 (HuB/H1N1), revealing that ADD1 knockout resulted in decreased virus titers (Figure 1d). We also examined the impact of ADD1 knockout on the replication of other host‐origin IAV strains and observed a similar reduction in replication of A/swine/Henan/F26/2017 (F26/H1N1), A/Puerto Rico/8‐SV14/1934 (PR8/H1N1), A/Hunan/42443/2015 (HuN/H1N1), and A/chicken/Hubei/115/2016 (115/H9N2) in ADD1‐KO cells (Figure 1e–h). Depletion of ADD1 using siRNA also reduced the replication of IAV (Figure 1i; Figure S1a, Supporting Information). Additionally, IAV infection could be rescued by expression of ADD1 in WT or ADD1‐KO cells (Figure 1j; Figure S1b, Supporting Information). The role of ADD1 in IAV replication in human A549 cells was also examined, and ADD1 was similarly required for IAV infection in A549 cells (Figure S1c–h, Supporting Information). These data confirmed that ADD1 acted as a pro‐viral host factor for IAV replication in different cell types.

### 2.2 ADD1 is Involved in the Early Stages of IAV Infection

**Figure 2:**
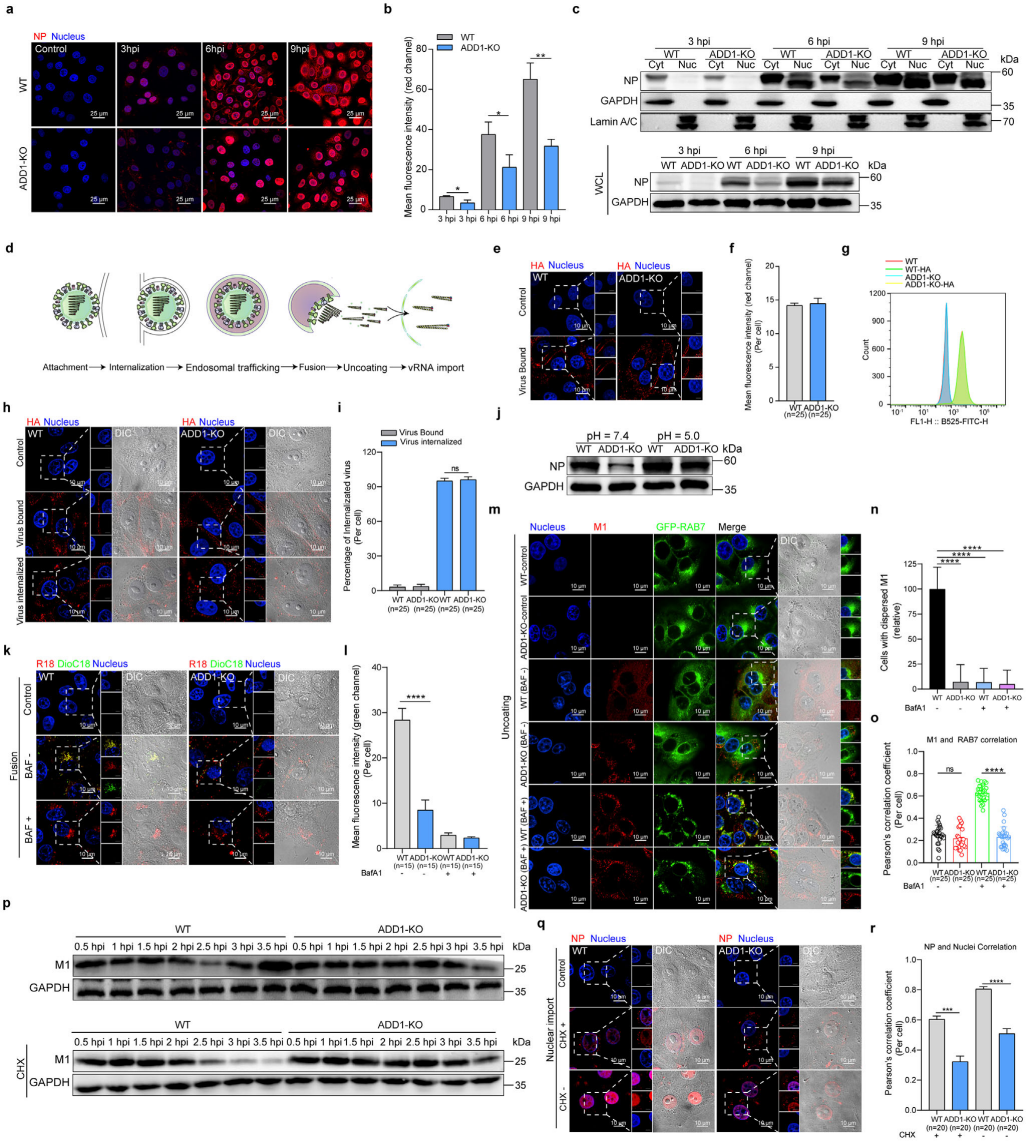
ADD1 affects IAV endosomal trafficking. a–c) WT or ADD1‐KO PK‐15 cells were infected with HuB/H1N1 (MOI = 10), and the amount and distribution of viral NP were detected by indirect immunofluorescence assay (a) or nuclear and cytoplasmic fractionation assay (c), with grayscale analysis for quantification (*n* = 3). NP (red), nucleus (blue). Scale bars represent 25 µm. Whole‐cell lysates (WCL). d) Schematics of early stages in the IAV replication cycle. e‐g) WT or ADD1‐KO PK‐15 cells were incubated with HuB/H1N1 (MOI = 50) at 4 °C for 1 h, and the amount of viral HA was detected by confocal microscopy (e) or flow cytometry (g) (*n* = 3). The values are indicated at the bottom of each image (*n* = 25 cells). HA (red), nucleus (blue). Scale bars represent 10 µm. The scale bars of the enlarged panel represent 5 µm. h, i) WT or ADD1‐KO PK‐15 cells were infected with HuB/H1N1 (MOI = 50) and subjected to binding assay or internalization assay (h). The amount of internalized virus was quantified (i), and values are indicated at the bottom of each image (*n* = 25 cells). HA (red), nucleus (blue). Scale bars represent 10 µm. The scale bars of the enlarged panel represent 5 µm. j) WT or ADD1‐KO PK‐15 cells were infected with HuB/H1N1 (MOI = 10) and subjected to an acid bypass assay. Viral NP expression was detected at 6 hpi, with grayscale analysis for quantification (*n* = 3). k, l) WT or ADD1‐KO PK‐15 cells were infected with dual‐labeled HuB/H1N1 (MOI = 50) to detect viral fusion at 1.5 hpi. Fluorescence intensity of DiOC18‐positive signal was quantified (l). The values are indicated at the bottom of each image (*n* = 15 cells). DiOC18 (green), R18 (red), nucleus (blue). Scale bars represent 10 µm. The scale bars of the enlarged panel represent 5 µm. m‐o) WT or ADD1‐KO PK‐15 cells were treated with CHX and infected with HuB/H1N1 (MOI = 50) to detect M1 staining at 2.5 hpi. The diffuse staining in M1‐positive cells was quantified (n) (*n* > 50 cells). The Pearson's correlation coefficient (PCC) of M1 and RAB7 was analyzed (o), and the values are indicated at the bottom of each image (*n* = 25 cells). M1 (red), nucleus (blue). Scale bars represent 10 µm. The scale bars of the enlarged panel represent 5 µm. p) WT or ADD1‐KO PK‐15 cells were treated with or without CHX and infected with HuB/H1N1 (MOI = 50) to detect M1 levels, with grayscale analysis for quantification (*n* = 3). q, r) WT or ADD1‐KO PK‐15 cells were infected with HuB/H1N1 (MOI = 20) to detect the nuclear translocation of NP by confocal microscopy. The Pearson's correlation coefficient (PCC) of NP and nuclei was analyzed (r). The values are indicated at the bottom of each image (*n* = 20 cells). NP (red), nucleus (blue). Scale bars represent 10 µm. The scale bars of the enlarged panel represent 5 µm. Data are presented as mean ± SD. Statistical analysis was performed using unpaired, two‐tailed Student's t‐test. *P < 0.05; **P < 0.01; ***P < 0.001; ****P < 0.0001; *ns*, not significant.

To further explore the phase of the IAV infection cycle in which ADD1 was implicated, the cellular distributions of the viral nucleoprotein (NP) in infected WT and ADD1‐KO cells were visualized. It was revealed that ADD1 knockout resulted in reduced NP expression at 3 hpi (**Figure** **2**a–c; Figure S2a,b, Supporting Information), indicating that ADD1 was involved in the early stages of IAV infection. The early successive steps of virus infection were further probed, and ADD1 did not function in either IAV attachment or internalization processes (Figure 2d–i). Following attachment and internalization, IAV exploits the endosomal pathway for cytoplasmic transport, which could be replaced by the fusion of viral envelope and plasma membranes at the cell surface through acid bypass treatment.^[^ ^18^, ^28^ ^]^ We found that viral NP expression level could be restored in acid bypass‐treated ADD1‐KO cells (Figure 2j; Figure S2c, Supporting Information), suggesting that ADD1 is involved in IAV endosomal trafficking processes. The defects in endosomal trafficking detected in ADD1‐KO cells predict that these cells should also exhibit inhibited viral membrane fusion, uncoating, and nuclear import. To test this possibility, dual‐wavelength imaging assays were performed to detect membrane fusion,^[^ ^29^ ^]^ and a significant reduction in the number of DiOC18 green fluorescence dots was observed in ADD1‐KO cells, indicating that viral membrane fusion was inhibited (Figure 2k,l). The subsequent uncoating process was further assessed by detecting the distribution of matrix protein 1 (M1) at 2.5 hpi. A limited amount of M1 diffusion and delayed M1 degradation was detected in ADD1‐KO cells compared with successful uncoating in almost all WT cells (Figure 2m–p; Figure S2d,e, Supporting Information). Additionally, the nuclear translocation of NP was also significantly hindered in ADD1‐KO cells (Figure 2q,r). In conclusion, the defects in viral membrane fusion, uncoating, and nuclear import in ADD1‐KO cells reinforce the conclusion that ADD1 is an important host factor during viral endosomal trafficking.

### 2.3 ADD1 Promotes the Trafficking of Internalized IAV from Clathrin‐Coated Vesicles to RAB5‐ and RAB7‐Positive Endosomes

**Figure 3:**
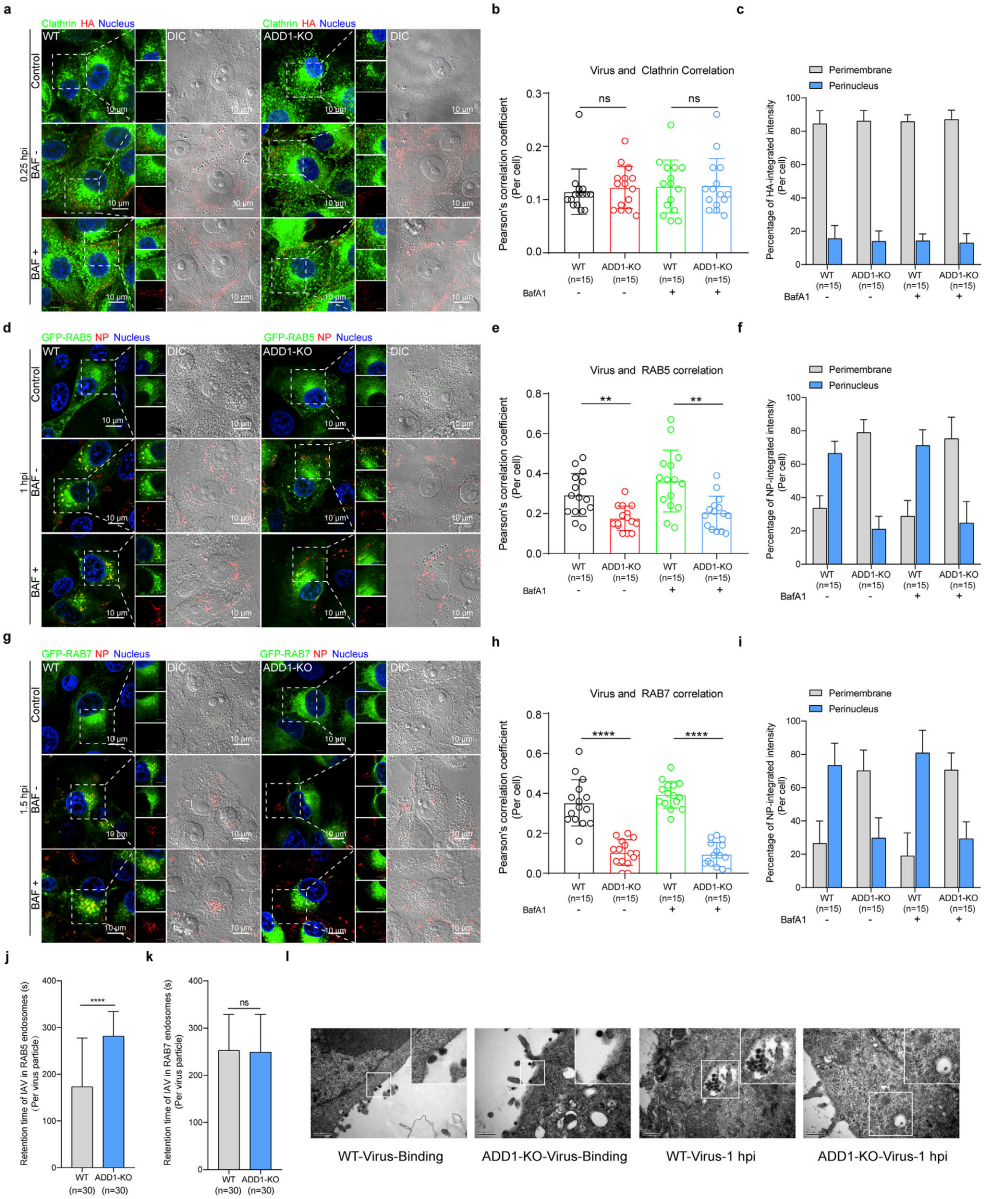
ADD1 promotes the trafficking of IAV from clathrin‐coated vesicles to RAB5 and RAB7‐positive endosomes. a–i) WT or ADD1‐KO PK‐15 cells were infected with HuB/H1N1 (MOI = 50). The co‐localization of viral HA (red) with Clathrin (green) at 0.25 hpi (a), viral NP (red) with GFP‐RAB5 (green) at 1 hpi (d), or viral NP (red) with GFP‐RAB7 (green) at 1.5 hpi (g) was acquired using the confocal microscope. The Pearson's correlation coefficient (PCC) of the virus and Clathrin (b), GFP‐RAB5 (e), or GFP‐RAB7 (h) was quantified, and the values are indicated at the bottom of each image (*n* = 15 cells). The perimembrane or perinuclear distribution of viruses at 0.25 hpi (c), 1 hpi (f), and 1.5 hpi (i) was analyzed, and the values are indicated at the bottom of each image (*n* = 15 cells). Scale bars represent 10 µm. Scale bars of the enlarged panel represent 5 µm. j, k) The time a virus particle spends within a RAB5 (j) or RAB7 (k) compartment was quantified, and the values are indicated at the bottom of each image (*n* = 30 virus particles). l) Transmission electron microscope images of HuB/H1N1 (MOI = 50) at 0 hpi and 1 hpi in WT or ADD1‐KO PK‐15 cells. Scale bars represent 0.5 µm. Data are presented as mean ± SD. Statistical analysis was performed using an unpaired, two‐tailed Student's t‐test. *P < 0.05; **P < 0.01; ***P < 0.001; ****P < 0.0001; *ns*, not significant.

To dissect how ADD1 governs viral endosomal trafficking, we probed the distinct phases of this process. We found that the colocalization of viral HA with clathrin heavy chain (a marker for clathrin‐coated vesicles) showed no difference, and the distribution of viral HA was unaffected after ADD1 depletion (**Figure** **3**a–c). In contrast, the colocalization of viral NP with GFP‐RAB5 (an early endosomal marker) and GFP‐RAB7 (a late endosomal marker) significantly decreased in ADD1‐KO cells. Interestingly, we noted that in ADD1‐KO cells, viral NP was primarily located beneath the plasma membrane region, with fewer viral NP presented in the perinuclear region compared to WT cells (Figure 3d–i). We next generated Quantum‐dot (QD)‐labeled IAVs and tracked their dynamic interactions with RAB5 and RAB7. Notably, virus‐containing RAB5 vesicles could move from the plasma membrane region to the perinuclear region, where they fused with the RAB7 endosomal membrane in WT cells. In contrast, in ADD1‐KO cells, numerous viruses accumulated in the plasma membrane region with limited trafficking to RAB5‐positive and RAB7‐positive endosomes (Videos S1 and S2, Supporting Information). Compared to WT cells, virus particles spent more time within RAB5‐positive endosomes, with no significant difference in residence time within RAB7‐positive endosomes in ADD1‐KO cells (Figure 3j,k). To further investigate whether there is a kinetic delay in IAV trafficking, we labeled IAV with QDs and conducted a 2.5‐h time‐course experiment. Our findings indicated that ADD1 knockout resulted in the majority of virus particles residing beneath the plasma membrane and inhibited the transport of IAV from the plasma membrane to the nucleus (Video S3, Supporting Information). Meanwhile, transmission electron microscopy (TEM) results showed that numerous virions were identified in late endosomes containing multiple intraluminal vesicles in WT cells, while the single particle predominantly resided in clathrin‐coated vesicles and early endosomal‐like structures in ADD1‐KO cells (Figure 3l). Taken together, these results indicated that ADD1 is required for a step involving the trafficking of internalized IAV from clathrin‐coated vesicles to RAB5 and RAB7‐positive endosomes.

### 2.4 ADD1 Depletion Results in a General Defect in Vesicular Trafficking

**Figure 4:**
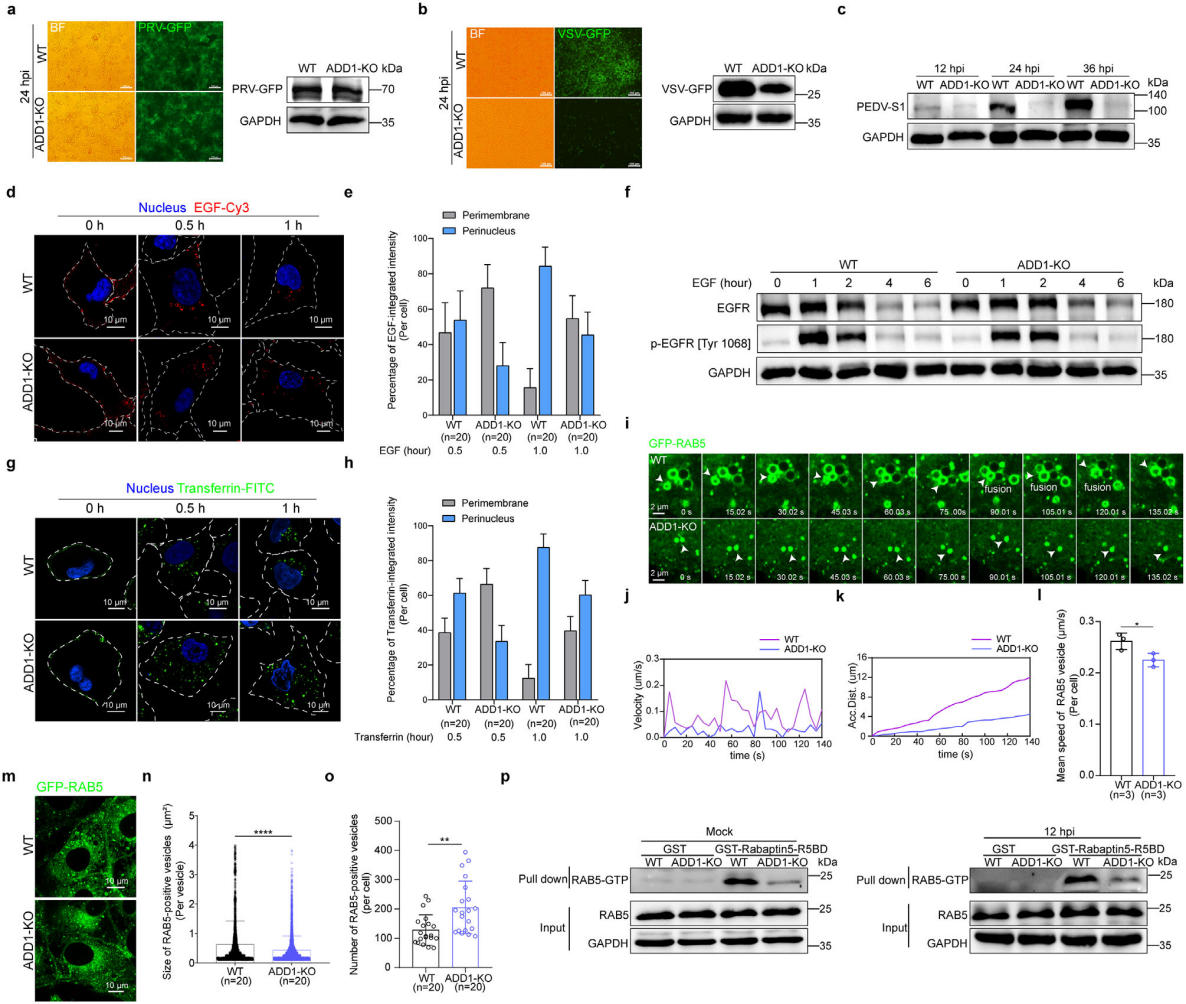
ADD1 depletion results in a general defect in vesicular trafficking. a–c) WT or ADD1‐KO PK‐15 cells were infected with PRV‐GFP (MOI = 0.1) (a), VSV‐GFP (MOI = 0.1) (b), or PEDV (MOI = 0.1) (c). The fluorescence of PRV‐GFP or VSV‐GFP was visualized by fluorescence microscopy, and the GFP level or PEDV‐S1 level was determined by immunoblot, with grayscale analysis for quantification (*n* = 3). Scale bars represent 100 µm. d, e, g, h) WT or ADD1‐KO A549 cells were stimulated with EGF (1 µg mL^−1^) (d) or transferrin (25 µg mL^−1^) (g) and subjected to fluorescence imaging. The perimembrane or perinuclear distribution of EGF (e) or transferrin (h) was analyzed, and the values are indicated at the bottom of each image (*n* = 20 cells). Scale bars represent 10 µm. f) WT or ADD1‐KO A549 cells were stimulated with EGF (100 ng mL^−1^), and the p‐EGFR and EGFR levels were assessed by immunoblot, with grayscale analysis for quantification (*n* = 3). i) Snapshots of the movement of a RAB5‐positive vesicle (pointed out with white arrows). j, k) Instantaneous velocity *vs*. time, accumulated distance vs. time plots of the RAB5‐positive vesicle. l) The mean speed of the RAB5‐positive vesicle was quantified, and the values are indicated at the bottom of each image (*n* = 3 cells). m‐o) WT or ADD1‐KO PK‐15 cells expressing GFP‐RAB5 were subjected to fluorescence imaging (m). The size (n) and the number (o) of RAB5‐positive vesicles were quantified, and the values are indicated at the bottom of each image (*n* = 20 cells). Scale bars represent 10 µm. p) WT or ADD1‐KO PK‐15 cells were infected with or without HuB/H1N1 (MOI = 1.0) and subjected to the GST‐Rabaptin5‐R5BD pulldown assay to detect RAB5‐GTP level, with grayscale analysis for quantification (*n* = 3). Data are presented as mean ± SD. Statistical analysis was performed using unpaired, two‐tailed Student's t‐test. *P < 0.05; **P < 0.01; ***P < 0.001; ****P < 0.0001; *ns*, not significant.

To determine whether ADD1 depletion specifically impacts IAV endosomal trafficking, we examined the effect of ADD1 depletion on the replication of other viruses. We found that ADD1 depletion inhibited the replication of vesicular stomatitis virus (VSV) and porcine epidemic diarrhea virus (PEDV), which utilize the intracellular vesicular transport system,^[^ ^30^, ^31^, ^32^ ^]^ and had no impact on the replication of pseudorabies virus (PRV), which enters cells through direct fusion with the plasma membrane^[^ ^33^, ^34^ ^]^ (**Figure** **4**a–c; Figure S3a–c, Supporting Information). Similar results were observed in other endocytic cargoes (EGF/EGFR and transferrin). ADD1 depletion resulted in the inhibition of EGFR degradation and transferrin recycling under uninfected conditions (Figure 4d–h; Figure S3d, Supporting Information), indicating that the defect in endosomal trafficking caused by ADD1 depletion is not virus‐specific but rather a broader impairment of general vesicular trafficking.

To delve into a deeper understanding of the role of ADD1 in endosomal transport, we investigated the dynamic movement of endosomes under uninfected conditions. In WT cells, RAB5‐positive endosomes moved rapidly from the plasma membrane region toward the perinuclear region, ultimately engaging in fusion events. Conversely, in ADD1‐KO cells, RAB5‐positive endosomes exhibited relatively random and slow movements beneath the plasma membrane (Figure 4i–l; Video S4, Supporting Information). Additionally, RAB5‐positive endosomes were smaller in size and more abundant in ADD1‐KO cells (Figure 4m–o). In contrast, there were no significant differences in the size, number, velocity, and distribution of RAB7‐positive endosomes between WT and ADD1‐KO cells (Figure S3e–k and Video S5, Supporting Information), suggesting that ADD1 depletion might disrupt RAB5‐positive endosome trafficking and fusion. Given that the disruption of RAB5 activity inhibits vesicle fusion,^[^ ^35^ ^]^ we next examined the activity of RAB5 by pulling down RAB5‐GTP with the RAB5 binding domain (R5BD) of rabaptin‐5 with or without IAV infection. The results showed that ADD1 knockout resulted in a decreased level of RAB5‐GTP regardless of IAV infection (Figure 4p; Figure S3l,m, Supporting Information).

### 2.5 Altered Cytoskeletal Organization by ADD1 Depletion Disrupts General Vesicular Trafficking

**Figure 5:**
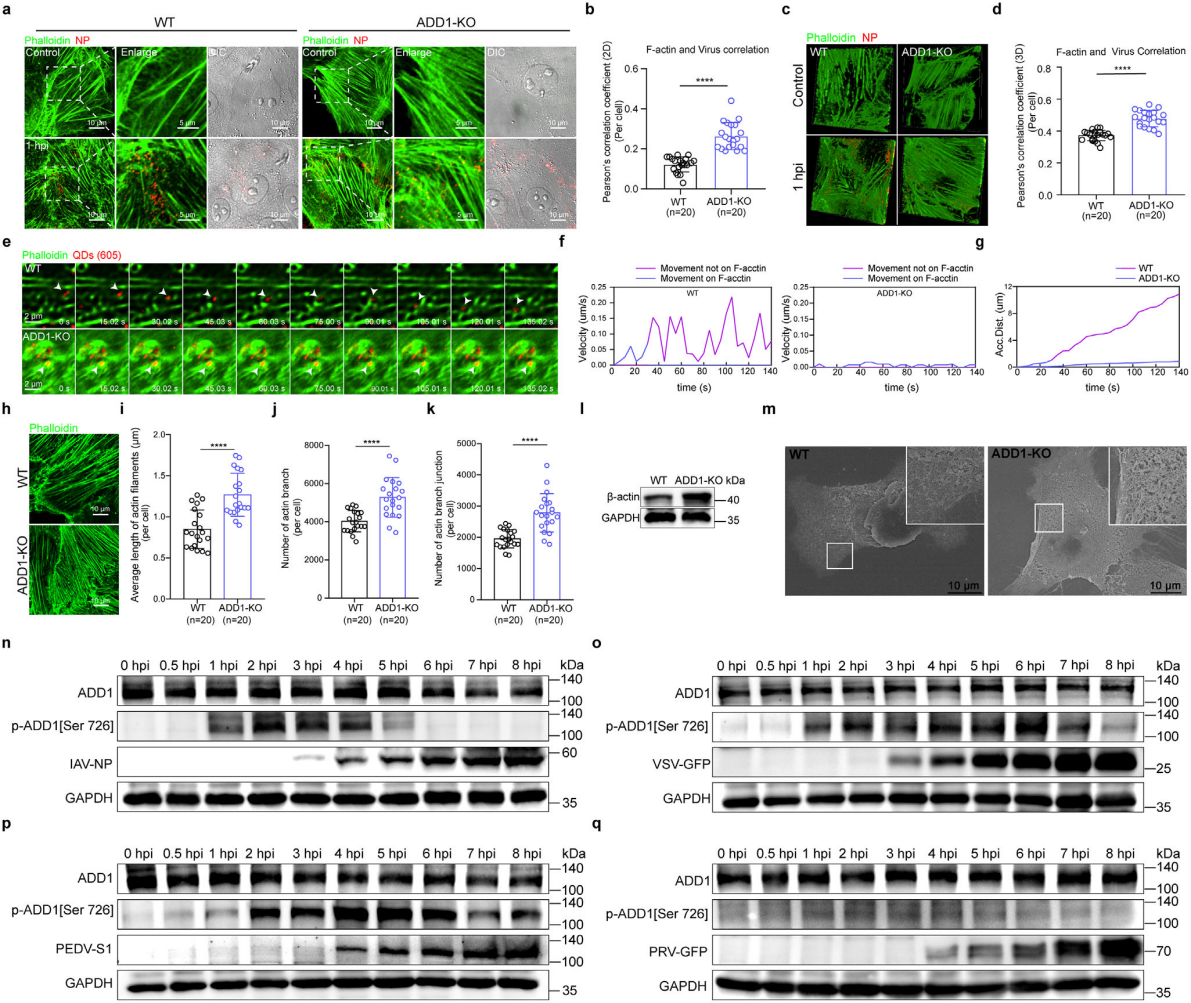
ADD1 depletion altered the actin network, resulting in the retention of IAVs on actin filaments. a‐d) WT or ADD1‐KO PK‐15 cells were infected with HuB/H1N1 (MOI = 50), and 2D images (a) or 3D images (c) showing the co‐localization of viral NP (red) with F‐actin (green) were acquired using a confocal microscope. The 2D images (b) or 3D images (d) of Pearson's correlation coefficient (PCC) of NP and F‐actin were quantified, and the values are indicated at the bottom of each image (*n* = 20 cells). Scale bars represent 10 µm. The scale bars of the enlarged panels represent 5 µm. e) Snapshots of a virus (pointed out with white arrows) moving on microfilaments. f, g) Instantaneous velocity *vs*. time (f), accumulated distance vs. time (g) plots of the tracked virus. h‐k) WT or ADD1‐KO PK‐15 cells were stained with phalloidin (green) to label F‐actin and subjected to fluorescence imaging. The average length of actin filaments (i), number of actin branches (j), and number of actin branch junctions (k) were quantified, and the values are indicated at the bottom of each image (*n* = 20 cells). Scale bars represent 10 µm. l) β‐actin expression in WT or ADD1‐KO PK‐15 cells was assessed by immunoblot, with grayscale analysis for quantification (*n* = 3). m) Scanning electron microscope images of the cytoskeleton in WT or ADD1‐KO PK‐15 cells. Scale bars represent 10 µm. n‐q) WT PK‐15 cells were infected with HuB/H1N1 (MOI = 10) (n), VSV‐GFP (MOI = 10) (o), PEDV (MOI = 10) (p), or PRV‐GFP (MOI = 10) (q), and the p‐ADD1 [Ser726] level was assessed by immunoblot, with grayscale analysis for quantification (*n* = 3). Data are presented as mean ± SD. Statistical analysis was performed using an unpaired, two‐tailed Student's t‐test. *P < 0.05; **P < 0.01; ***P < 0.001; ****P < 0.0001; *ns*, not significant.

As an actin‐binding protein, ADD1 may regulate viral vesicular trafficking by modulating actin remodeling. Consistently, ADD1‐KO cells exhibited increased accumulation of IAV in actin‐rich regions (**Figure** **5**a–d; Video S6, Supporting Information). Additionally, tracking the movement of QD‐labeled viruses revealed that QD‐labeled IAV in WT cells could rapidly escape actin filaments at a speed of up to 0.15 µm s^−1^, accumulating a distance of ≈1.03 µm on F‐actin and ≈9.85 µm away from F‐actin. In contrast, numerous QD‐labeled viruses in ADD1‐KO cells moved slowly on F‐actin at a speed of ≈0.006 µm s^−1^. The viruses appeared to be trapped and unable to move away from F‐actin, and the accumulated distance in this period was ≈0.84 µm (Figure 5e–g; Video S7, Supporting Information). To ascertain whether the slow movement of IAV in ADD1‐KO cells is due to a defect in the microtubule system, we next examined the movement of IAV on microtubules. The results showed that the length and amount of tubulin, the velocity and accumulated distance of IAV on microtubules were not affected in ADD1 knockout cells (Figure S4 and Video S8, Supporting Information), indicating that ADD1 affects the microfilament‐dependent transport of viruses rather than microtubule‐dependent transport. Similar to the retention of viruses on F‐actin after ADD1 deletion, there was an increased accumulation of p‐EGFR on actin filaments in ADD1‐KO cells with EGF stimulation (Figure S5a,b, Supporting Information). Additionally, by analyzing the architecture of endosomal actin through high‐resolution images, we found that there were more actin branches around the RAB5‐positive vesicles, with more small endosomes distributed along longer F‐actin filaments in ADD1‐KO cells compared to WT cells (Figure S5c,d, Supporting Information). By analyzing the microfilament distribution, we found a significant increase in the length, number of branches, number of branch junctions, and total protein of actin in ADD1‐KO cells (Figure 5h–l; Figure S5e, Supporting Information). Additionally, cytoskeletal fibers were irregular and disordered, primarily appearing branched and elongated in ADD1‐KO cells, while in WT cells, cytoskeletal fibers exhibited varying thicknesses, forming an interwoven network structure with subtle swelling at the branch sites (Figure 5m). These results demonstrated that ADD1 depletion altered cytoskeletal organization, increased the density of F‐actin around RAB5‐positive endocytic vesicles, and resulted in the accumulation of endocytosed viruses and cargo in actin‐rich regions.

To gain deeper insights into the dynamic remodeling of the actin network, a time‐course experiment was conducted with or without the R18‐labeled IAV challenge. The results showed that IAV infection significantly increased the dynamic remodeling of F‐actin in WT cells, while actin dynamics in ADD1‐KO cells were inhibited (Figure S6a and Videos S9 and S10, Supporting Information). Previous studies have shown that phosphorylation of ADD1 at Ser726 disrupts its actin‐capping activity, destabilizing the spectrin‐actin network and promoting actin filament depolymerization and reorganization.^[^ ^20^, ^21^, ^24^, ^25^, ^26^ ^]^ To determine whether IAV infection induces ADD1 phosphorylation to drive actin remodeling, we examined ADD1 phosphorylation in IAV‐infected cells and observed an upregulation of ADD1 phosphorylation at Ser726 at 1–4 hpi (Figure 5n; Figure S6b, Supporting Information). Since phosphorylation of ADD1 at Ser726 induces its dissociation from F‐actin and subsequent caspase‐3‐mediated degradation,^[^ ^36^ ^]^ we examined the subcellular localization and expression levels of ADD1 during IAV infection. As expected, IAV infection reduced plasma membrane‐associated ADD1 and caused a time‐dependent decrease in ADD1 protein levels with escalating IAV doses (Figure S6c–e, Supporting Information). These findings demonstrated that IAV could mediate actin remodeling by inducing the phosphorylation of ADD1 at Ser726.

To determine whether phosphorylation of ADD1 at Ser726 is necessary for actin remodeling and IAV trafficking, we stably expressed either WT ADD1 (Ser726) or a phospho‐deficient mutant (S726A) in ADD1‐KO cells. Compared to WT ADD1, the S726A mutant failed to effectively reduce actin‐network density (Figure S6f–i, Supporting Information), confirming that Ser726 phosphorylation is essential for actin filament depolymerization and reorganization. Furthermore, the S726A mutant could not effectively restore IAV trafficking and replication (Figure S6j–l, Supporting Information), indicating that Ser726 phosphorylation was critical for IAV transport. Given that ADD1 deletion causes a general defect in vesicular trafficking, we further investigated whether both endocytic viruses and cargo could induce ADD1 phosphorylation at Ser726 to facilitate F‐actin rearrangement. The results showed that endocytic viruses like VSV and PEDV induced an upregulation of ADD1 phosphorylation at Ser726 during early infection. However, PRV, a membrane fusion virus, could not induce an upregulation of ADD1 phosphorylation (Figure 5o–q; Figure S7a–c, Supporting Information). Similarly, endocytic cargoes like EGF triggered an increase in ADD1 phosphorylation at Ser726 (Figure S7d, Supporting Information). Together, these findings indicated that both endocytic viruses and cargo triggered ADD1 phosphorylation at Ser726 to mediate actin filament depolymerization and reorganization for their effective transport.

### 2.6 Loss of ADD1 Increases the Density of Arp2/3‐Mediated Actin Branches and Captures the Virus on Actin Branches

**Figure 6:**
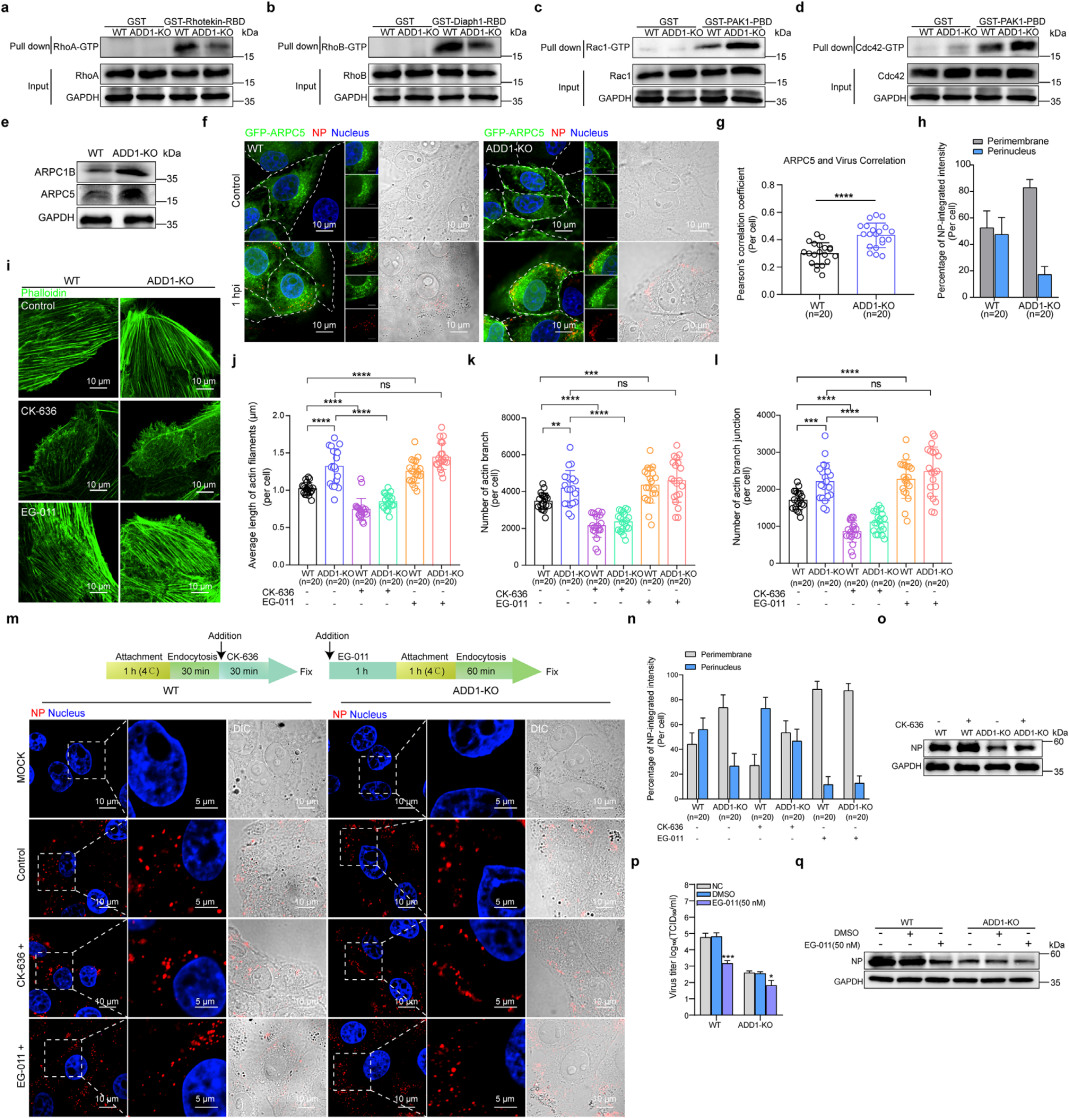
Loss of ADD1 increases actin branch density and captures the virus in the actin branch. a‐d) GST or GST‐Rhotekin‐RBD (a), GST‐Diaph1‐RBD (b), and GST‐PAK1‐PBD (c, d) recombinant proteins were incubated with GST Resin. The bead‐protein complexes were then incubated with lysates of WT or ADD1‐KO PK‐15 cells. The precipitated complexes were analyzed by immunoblot, with grayscale analysis for quantification (*n* = 3). e) The expression level of ARPC1B and ARPC5 in WT or ADD1‐KO PK‐15 cells was assessed by immunoblot, with grayscale analysis for quantification (*n* = 3). f‐h) WT or ADD1‐KO PK‐15 cells were infected with HuB/H1N1 (MOI = 50), and images of the co‐localization of viral NP (red) with GFP‐ARPC5 (green) were acquired using a confocal microscope (f). The Pearson's correlation coefficient (PCC) of NP and GFP‐ARPC5 was quantified (g). The perimembrane or perinuclear distribution of NP was analyzed (h), and the values are indicated at the bottom of each image (*n* = 20 cells). Scale bars represent 10 µm. The scale bars of the enlarged panels represent 5 µm. i‐l) WT or ADD1‐KO cells were treated with CK‐636 (100 µM) or EG‐011 (50 nM) and stained with phalloidin (green), followed by fluorescence imaging (i). The average length of actin filaments (j), number of actin branches (k), and number of actin branch junctions (l) were quantified, and the values are indicated at the bottom of each image (*n* = 20 cells). Scale bars represent 10 µm. m, n) WT or ADD1‐KO cells were treated with CK‐636 (100 µM) or EG‐011 (50 nM) and infected with HuB/H1N1 as indicated in the schematic, followed by fluorescence imaging (m). The perimembrane or perinuclear distribution of NP (red) was analyzed (n), and the values are indicated at the bottom of each image (*n* = 20 cells). Scale bars represent 10 µm. o) WT or ADD1‐KO PK‐15 cells were treated with or without CK‐636 (100 µM) for 20 min after virus endocytosis and NP expression was assessed by immunoblot at 6 hpi, with grayscale analysis for quantification (*n* = 3). p, q) WT or ADD1‐KO PK‐15 cells were treated with or without EG‐011 (50 nM) and then infected with HuB/H1N1 (MOI = 0.01) to assess viral titers (p) and viral NP expression (q) at 12 hpi, with grayscale analysis for quantification (*n* = 3). Data are presented as mean ± SD. Statistical analysis was performed using unpaired, two‐tailed Student's t‐test. *P < 0.05; **P < 0.01; ***P < 0.001; ****P < 0.0001; *ns*, not significant.

Given that cytoskeletal dynamics are orchestrated by three canonical Rho GTPases (Rho, Rac1, and Cdc42),^[^ ^37^ ^]^ we next examined the activity of Rho GTPases by pulling down Rho GTPase with relevant effector molecules. GST pull‐down analysis revealed a significant decrease in the activity of Rho (RhoA and RhoB) and a marked increase in the activity of Rac1 and Cdc42 in ADD1‐KO cells (**Figure** **6**a–d; Figure S8a–d, Supporting Information). Since Rac1‐GTP and Cdc42‐GTP promote Arp2/3‐mediated polymerization of actin branches,^[^ ^38^, ^39^ ^]^ we further analyzed Arp2/3 complex components. Notably, the expression of ARPC1B and ARPC5, key subunits of the Arp2/3 complex, was upregulated after ADD1 depletion (Figure 6e; Figure S8e, Supporting Information). Additionally, Cortactin, a stabilizer of actin branches, showed increased expression in ADD1‐KO cells (Figure S8f, Supporting Information). These findings demonstrated that ADD1 knockout led to Arp2/3 complex activation, resulting in the formation of dense actin branch networks. To determine whether viral entrapment on actin filaments in ADD1‐KO cells results from Arp2/3 complex activation, we examined the colocalization of RAB5 and viral NP with ARPC5 (a subunit of the Arp2/3 complex labeling actin branches). Indeed, more RAB5 and viral NP were associated with ARPC5 in ADD1‐KO cells compared to WT cells (Figure 6f–h; Figure S8g,h, Supporting Information). To determine whether the increased Arp2/3‐mediated actin branch formation induced by ADD1 deletion inhibited IAV trafficking, WT and ADD1‐KO cells were treated with CK‐636 (an Arp2/3 complex inhibitor) or EG‐011 (an actin branch polymerization activator).^[^ ^40^ ^]^ It was found that CK‐636 treatment decreased the length, number, and junctions of actin branches, while EG‐011 treatment increased these parameters (Figure 6i–l). Additionally, viral replication was partially restored, and the viruses partially overcame the actin branch constraint and relocated to the perinuclear region in ADD1‐KO cells following treatment with CK‐636 (Figure 6m–o; Figure S8i–l, Supporting Information). In contrast, treatment with EG‐011 resulted in the inhibition of IAV replication, and the majority of virus resided beneath the plasma membrane (Figure 6m,n,p,q; Figure S8m, Supporting Information). Collectively, ADD1 knockout increased the density of Arp2/3‐mediated actin branches, resulting in the retention of IAV on branched actin networks.

To determine whether the defect in IAV transport from clathrin‐coated vesicles to RAB5‐ and RAB7‐positive compartments in ADD1‐KO cells results from dense actin branching, we treated the cells with EG‐011 to promote actin branch polymerization and examined distinct phases of IAV trafficking. We found that EG‐011 treatment did not alter the co‐localization of viral HA with clathrin heavy chain (Figure S9a,b, Supporting Information). However, the co‐localization of viral NP with GFP‐RAB5 and GFP‐RAB7 was significantly reduced (Figure S9c–f, Supporting Information). These findings suggested that the dense actin branches in ADD1‐KO cells hindered IAV transport from clathrin‐coated vesicles to RAB5‐ and RAB7‐positive compartments. Since efficient intracellular vesicular transport is crucial for IAV trafficking, we next examined whether EG‐011‐induced actin branch polymerization also affects other viruses with distinct entry mechanisms. Similar to the effect of ADD1 knockout, EG‐011‐induced actin branch polymerization had no impact on the replication of PRV (Figure S9g,h, Supporting Information), which enters host cells via direct plasma membrane fusion, but significantly suppressed the replication of VSV and PEDV, both of which rely on intracellular vesicular transport for viral entry and trafficking (Figure S9i–k, Supporting Information).

To further determine the effect of EG‐011 treatment on IAV replication in vivo, 4‐ to 6‐week‐old female BALB/c mice were intraperitoneally injected with EG‐011, or an equal volume of vehicle (the solvent), followed by challenging with 30 pfu of HuN/H1N1 (Figure S10a, Supporting Information). Compared to the solvent group (Control), mice treated with EG‐011 exhibited a notable reduction in weight loss and an increase in survival (Figure S10b,c, Supporting Information). Furthermore, the lungs of EG‐011‐treated mice exhibited significantly reduced viral titers and diminished virus‐induced lung tissue damage on day 3 and day 5 post‐challenge (Figure S10d,e, Supporting Information). Immunofluorescence staining against the viral nucleoprotein revealed a lower presence of viral antigen in the lungs of the EG‐011‐treated group (Figure S10f, Supporting Information). Thus, actin branch elongation and formation contributed to a protective effect against IAV challenge in vivo.

### 2.7 ADD1 Promotes IAV Uncoating by Regulating the Activity of Myosin IIB

**Figure 7:**
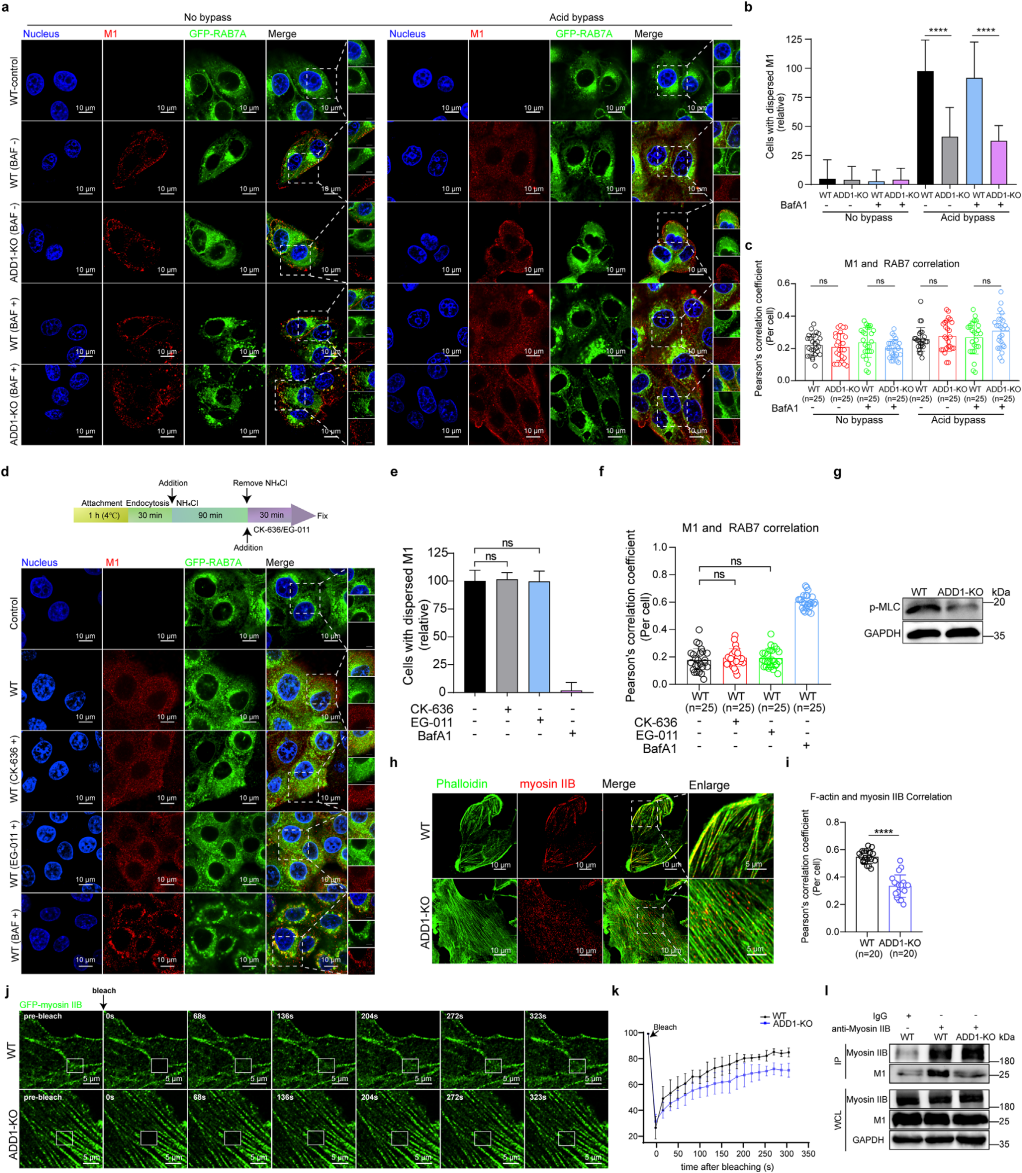
ADD1 facilitates IAV uncoating by modulating the activity of myosin IIB. a–c) WT or ADD1‐KO PK‐15 cells were infected with HuB/H1N1 (MOI = 50) and subjected to the acid bypass M1‐uncoating assay (a). The diffuse staining in M1‐positive cells was quantified (b) (*n* > 50 cells), and the Pearson's correlation coefficient (PCC) of M1 and RAB7 was analyzed (c). The values are indicated at the bottom of each image (*n* = 25 cells). GFP‐RAB7 (green), M1 (red), nucleus (blue). Scale bars represent 10 µm. The scale bars of the enlarged panel represent 5 µm. d‐f) WT cells were infected with HuB/H1N1 (MOI = 50) and treated with NH_4_Cl for 1.5 h to allow virus particles to accumulate in the late endosomes. NH_4_Cl was subsequently removed, and the cells were treated with CK‐636 (100 µM), EG‐011 (50 nM), or bafilomycin A1 (100 nM) for 0.5 h, followed by fluorescence imaging (d). The diffuse staining in M1‐positive cells was quantified (e) (*n* > 50 cells), and the Pearson's correlation coefficient (PCC) of M1 and RAB7 was analyzed (f). The values are indicated at the bottom of each image (*n* = 25 cells). GFP‐RAB7 (green), M1 (red), nucleus (blue). Scale bars represent 10 µm. g) p‐MLC expression in WT or ADD1‐KO PK‐15 cells was assessed by immunoblot, with grayscale analysis for quantification (*n* = 3). h, i) WT or ADD1‐KO A549 cells were stained with phalloidin (green) to label F‐actin, immunostained with antibodies against myosin IIB (red), and subjected to fluorescence imaging (h). The Pearson's correlation coefficient (PCC) of myosin IIB and F‐actin was quantified (i) (*n* = 20 cells). Scale bars represent 10 µm. j) Snapshots of GFP‐myosin IIB expression in WT or ADD1‐KO A549 cells before and after photobleaching. Scale bars represent 5 µm. k) Averaged FRAP curves showing recovery kinetics of GFP‐myosin IIB in WT and ADD1‐KO A549 cells (*n* = 3 cells). l) WT or ADD1‐KO PK‐15 cells were infected with HuB/H1N1 (MOI = 50) and subjected to the acid bypass M1‐uncoating assay. The cells were lysed at 15 min after acid bypass treatment. The lysate was immunoprecipitated using anti‐myosin IIB antibody to detect M1 and myosin IIB, and immunoprecipitated M1 was quantified by grayscale analysis (*n* = 3). Data are presented as mean ± SD. Statistical analysis was performed using unpaired, two‐tailed Student's t‐test. *P < 0.05; **P < 0.01; ***P < 0.001; ****P < 0.0001; *ns*, not significant.

In acid bypass‐treated ADD1‐KO cells, viral NP expression could be partially restored (Figure 2j), suggesting that, in addition to its involvement in viral endosomal trafficking, ADD1 might also function during the viral uncoating process. To exclude the effect of endosomal trafficking on viral uncoating, an acid bypass assay was performed to synchronize the penetration of cores into the cytosol. In ADD1‐KO cells, M1 uncoating was reduced by 60% compared to WT cells following acid bypass treatment (**Figure** **7**a–c). Notably, despite the observed impairment in M1 uncoating efficiency, co‐immunoprecipitation assays failed to detect the interaction between ADD1 and M1 (Figure S11a, Supporting Information), suggesting that ADD1 did not directly drive IAV uncoating. Since ADD1 deletion resulted in the increase of the actin branch, the observed M1 dispersal defect upon viral fusion might be due to the inhibitory effect of highly dense branched actin. To test this, we treated the cells with EG‐011 or CK‐636 to determine whether the actin branch would affect viral uncoating under physiological conditions (Figure 7d). No statistical difference was found in M1 dispersal among the untreated cells, the CK‐636‐treated cells, and the EG‐011‐treated cells (Figure 7e,f), suggesting that the effects of ADD1 during viral uncoating were independent of actin branch signaling.

Since myosin IIB on stress fibers generates opposing physical forces to break apart the viral capsid,^[^ ^18^ ^]^ its activity is regulated by the RhoA GTPase and the downstream phosphorylation of myosin light chain (MLC), which leads to the activation of its ATPase activity and promotes its binding to F‐actin, thereby driving force generation.^[^ ^41^, ^42^, ^43^, ^44^ ^]^ We found that ADD1 knockout led to a decrease in RhoA‐GTP levels (Figure 6a). To further determine whether ADD1 affects IAV uncoating by influencing myosin IIB activity, we next investigated the effects of ADD1 knockout on the downstream phosphorylation of MLC. We found that ADD1 knockout decreased MLC phosphorylation (Figure 7g; Figure S11b, Supporting Information), which inhibited the myosin IIB‐F‐actin interaction (Figure 7h,i), thereby impairing myosin IIB‐mediated force generation on stress fibers. Furthermore, fluorescence recovery after photobleaching (FRAP) experiments showed that ADD1 knockout prolonged the fluorescence recovery time of myosin IIB (Figure 7j,k; Video S11, Supporting Information), indicating weakened dynamic binding of myosin IIB to F‐actin. With acid bypass treatment, the interaction between myosin IIB and M1 was attenuated in ADD1‐KO cells compared to WT cells (Figure 7l; Figure S11c, Supporting Information). Together, these results suggested that ADD1 knockout disrupted the RhoA/MLC/myosin IIB axis, reducing the dynamic generation of myosin IIB‐mediated physical force on stress fibers required for IAV uncoating. ADD1 binds to F‐actin via its tail domain.^[^ ^20^, ^21^ ^]^ To further investigate whether the regulation of myosin IIB by ADD1 depends on its actin‐binding activity, we generated an ADD1 mutant lacking the tail domain (ADD1‐Δtail) and examined its regulatory effects on myosin IIB. The results showed that ADD1‐Δtail was unable to promote the phosphorylation of MLC, and the co‐localization of myosin IIB with actin was not restored upon expression of ADD1‐Δtail in ADD1 knockout cells (Figure S11d–f, Supporting Information). These results indicated that the regulation of myosin IIB by ADD1 depended on its actin‐binding activity.

### 2.8 ADD1 Promotes IAV Infection In Vivo

**Figure 8:**
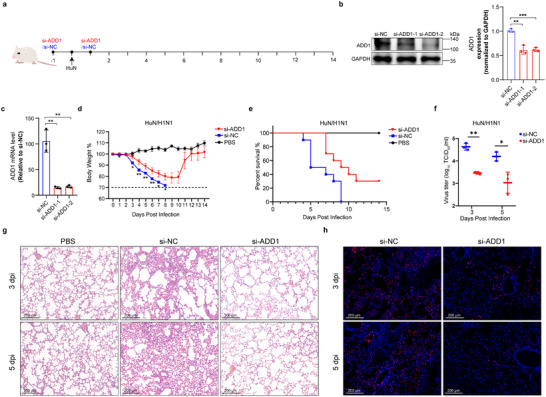
ADD1 knockdown attenuates IAV infection in vivo. a) Schematic of siRNA treatment and HuN/H1N1 infection in a mouse experimental model. b, c) The expression levels of ADD1 in si‐NC and si‐ADD1 treated mice were analyzed by immunoblot (b) or qRT‐PCR (c), with grayscale analysis for quantification (*n* = 3). d) Percentage change in body weight of si‐NC and si‐ADD1 treated mice. The dotted line represents 70% of the initial weight. Data are mean ± SEM (*n* = 10 mice). e) Survival rates of si‐NC and si‐ADD1 treated mice (*n* = 10 mice, with a log‐rank test [Mantel Cox]). f) Viral titers in the lungs of si‐NC and si‐ADD1 treated mice were measured on days 3 and 5. Data are mean ± SD (*n* = 3 mice). g) The lungs of si‐NC and si‐ADD1 treated mice were stained with (H&E) for histopathological diagnostics. Scale bars represent 200 µm. h) Representative immunofluorescence images of viral NP (red) in lung alveolar epithelial cells. Scale bars represent 200 µm. *P < 0.05; **P < 0.01; ***P < 0.001; ****P < 0.0001; *ns*, not significant.

As ADD1 deficiency exhibited antiviral effects on IAV infection in vitro, we next examined the role of ADD1 in IAV infection in vivo. The mice were nasally instilled with chemically cholesterol‐conjugated and 2′‐OME‐modified siRNA targeting ADD1 (si‐ADD1) or negative control siRNA (si‐NC) and challenged with 30 pfu of HuN/H1N1 (**Figure** **8**a). The knockdown efficiency of si‐ADD1 in mice was evaluated and revealed significantly reduced ADD1 expression (Figure 8b,c). Weight loss and survival rates were monitored in each group for 14 days post‐infection. Compared to the control mice, si‐ADD1‐treated mice suffered less weight loss and had a higher survival rate, with a protective rate of 30% against the IAV challenge (Figure 8d,e). Additionally, viral titers significantly decreased in the si‐ADD1‐treated mice compared to the control mice on day 3 and day 5 post‐challenge (Figure 8f). Hematoxylin and eosin (H&E) staining of lungs in the control mice showed alveolar wall thickening, alveolar capillaries hyperemia, and severe inflammatory cellular infiltration, while the si‐ADD1‐treated mice displayed only modest denudation of the alveolar septum, with slight tissue damage and inflammatory cell infiltration (Figure 8g). Immunofluorescence staining against IAV nucleoprotein showed less viral antigen in the lungs of si‐ADD1‐treated mice compared to control mice (Figure 8h). Altogether, the results suggested that ADD1 knockdown conferred a certain level of protection to mice against lethal IAV challenge.

## 3 Discussion

**Figure 9:**
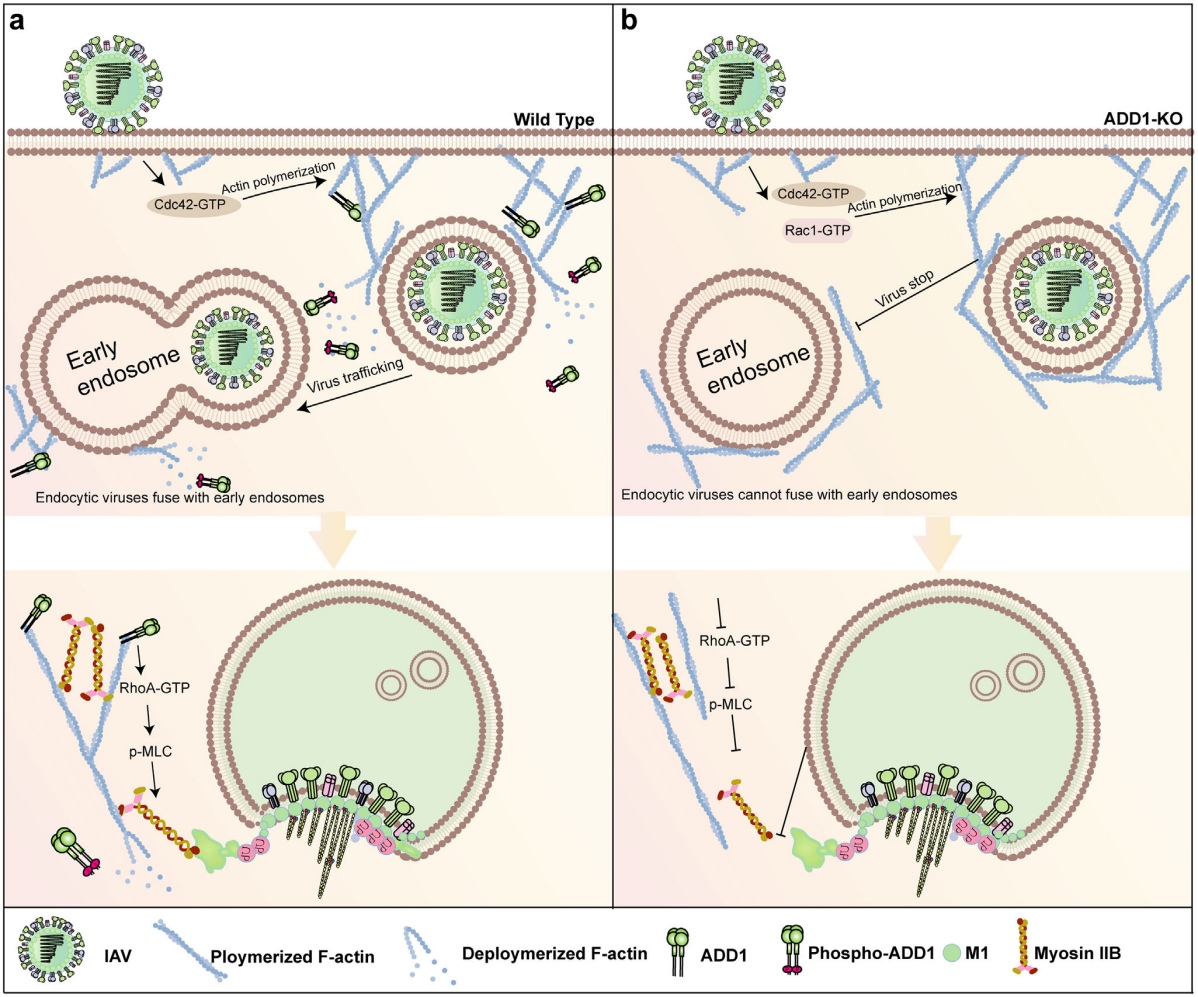
Graphical summary of IAV endocytic trafficking and uncoating in the presence or absence of ADD1. a) ADD1 coordinates actin rearrangement, facilitating the fusion of virus‐containing vesicles with early endosomes and promoting viral detachment from actin filaments, allowing rapid movement along microtubules. Once the viral envelope fuses with the endosomal membrane, ADD1 facilitates viral uncoating by modulating the RhoA/MLC/myosin IIB axis. b) ADD1 deletion promotes actin branch polymerization, leading to the retention of virus‐containing vesicles in the actin network and inhibiting viral uncoating by perturbing the RhoA/MLC/myosin IIB axis.

Our results elucidate the pivotal role of ADD1 in regulating F‐actin dynamics and myosin IIB activity during IAV endocytic trafficking and uncoating. IAV infection activates Cdc42 to assemble actin filaments around endocytic vesicles, leading to the formation, constriction, and internalization of IAV‐containing vesicles.^[^ ^45^, ^46^ ^]^ Subsequent phosphorylation of ADD1 at Ser726 triggers the depolymerization and rearrangement of actin branches. The depolymerization of actin branches around vesicles facilitates the fusion of the nascent vesicle with the early endosome and their attachment to microtubules for rapid movement. Upon viral envelope fusion with the endosomal membrane, ADD1 facilitates viral uncoating by modulating the RhoA/MLC/myosin IIB axis (**Figure** **9**a). ADD1 depletion increases the activity of Rac1 and Cdc42, resulting in the elongation and densification of actin branches around endocytic vesicles, which trap the virus in branched F‐actin networks. In addition, viral uncoating is hindered due to the perturbation of the RhoA/MLC/myosin IIB axis (Figure 9b).

While several host factors involved in IAV endosomal trafficking have been identified, little is known about the transport of IAV from clathrin‐coated vesicles to early endosomes. Previous studies have reported that cytohesin 2 (CYTH2) and transforming acidic coiled‐coil containing protein 3 (TACC3) regulate the transport of IAV from early to late endosomes.^[^ ^47^, ^48^ ^]^ Additionally, transcription factor E3 (TFE3) and nuclear receptor coactivator 7 (NCOA7) influence viral membrane fusion by modulating the acidification of late endosomes.^[^ ^49^, ^50^ ^]^ In this study, we found that IAV infection upregulated ADD1 phosphorylation at Ser726, which reduced the stability and density of the actin network, thereby facilitating the fusion of clathrin‐coated vesicles with early endosomes. This suggests that actin dynamics, a key factor in this process, are fundamental for IAV entry and endosomal trafficking.

Studies have shown that F‐actin polymerization is required for vesicle‐packaged virus deformation, scission, and detachment from the plasma membrane.^[^ ^10^, ^12^, ^45^ ^]^ However, despite its well‐established role in the initial steps of viral internalization, its involvement in subsequent viral trafficking remains elusive. Here, we found that virus‐induced dynamic rearrangement of F‐actin followed a pattern of initial polymerization followed by depolymerization (Figure S6a and Videos S9 and S10, Supporting Information). ADD1 knockout disrupts the recruitment of spectrin to the growing filamentous actin initiated by Arp2/3 to form stable spectrin‐actin networks. The free actin filaments are targeted by cofilin, resulting in their severing and depolymerization.^[^ ^22^, ^51^ ^]^ However, the filaments newly polymerized from cofilin‐severed barbed ends are ATP‐rich filaments that promote the nucleation and branching activity of the Arp2/3 complex.^[^ ^52^ ^]^ Without ADD1 capping and recruiting spectrin to stabilize actin filaments, actin monomers continuously polymerize into filamentous actin in an uncontrolled manner. This, in turn, activates actin polymerization activators (Rac1 and Cdc42), further propelling Arp2/3‐mediated actin branch polymerization, ultimately trapping the virus in branched F‐actin networks and disrupting internalized IAV trafficking (Figure 6; Figure S8, Supporting Information). Therefore, actin filaments around the virus‐containing vesicle perform dual functions in virus entry—promoting the formation of internalized IAV‐containing vesicles and acting as a physical barrier to inhibit the trafficking of internalized IAV from nascent vesicles to RAB5‐ and RAB7‐positive endosomes.

The protein kinase C (PKC) pathway is activated during virus endosomal trafficking.^[^ ^53^, ^54^ ^]^ As a substrate for PKC, PKC‐mediated phosphorylation of ADD1 at Ser726 destabilizes the spectrin‐F‐actin network and promotes actin filament depolymerization and reorganization.^[^ ^55^, ^56^ ^]^ Viruses (IAV, VSV, and PEDV) and endocytic cargoes (EGF/EGFR), which rely on intracellular vesicular transport for entry, possessed the capability to upregulate the phosphorylation of ADD1. In contrast, PRV, which enters host cells via direct plasma membrane fusion, could not induce an upregulation of ADD1 phosphorylation (Figure 5n–q; Figure S7, Supporting Information). Thus, endocytic viruses or cargoes might trigger the PKC/ADD1 signaling pathway, decreasing the density of actin branches around endocytic vesicles to facilitate their attachment to microtubules for rapid movement toward the perinuclear region. This was confirmed by observations that treatment with Gö6976 (a PKC inhibitor) or EG‐011 inhibited IAV trafficking (Figure 6m; Figure S9a–f, Supporting Information).^[^ ^54^ ^]^ Additionally, both EG‐011 treatment and ADD1 knockdown attenuated IAV replication in vivo (Figure 8; Figure S10, Supporting Information). Consequently, PKC/ADD1/F‐actin is an attractive target for the development of antiviral drugs to prevent multiple virus infections utilizing intracellular vesicular transport system.

Similar to ADD1, colchicine (a microtubule‐targeting drug) has been shown to effectively inhibit the replication of various viruses, including respiratory syncytial virus (RSV), human immunodeficiency virus (HIV), and IAV, by destabilizing microtubules and accelerating their disassembly.^[^ ^57^, ^58^ ^]^ Although colchicine was used for clinical treatment during the COVID‐19 pandemic, it exhibits relatively high cytotoxicity and is therefore not currently used as an antiviral drug in clinical applications.^[^ ^57^, ^59^ ^]^ In contrast to colchicine, ADD1 knockout promotes actin polymerization rather than disassembly, making it a potential broad‐spectrum antiviral target with reduced cytotoxicity, particularly for combating newly emerging viruses.

The actin cytoskeleton comprises branched actin filaments and stress fibers, each fulfilling distinct roles during IAV infection. Our study reveals that branched actin primarily orchestrates viral endocytosis and subsequent trafficking but is dispensable for IAV uncoating (Figure 7d–f). In contrast, myosin IIB associated with stress fibers generates opposing mechanical forces to disrupt the viral capsid, facilitating uncoating.^[^ ^18^ ^]^ Moreover, we identify ADD1 as a key regulator of this process, promoting IAV uncoating via the RhoA/MLC/myosin IIB signaling axis (Figures 6a and 7g–l; Video S11, Supporting Information). These findings provide mechanistic insights into how actin dynamics govern the early stages of IAV infection.

In this work, we dissected the role of ADD1 in IAV endosomal trafficking and uncoating by utilizing QDs‐based virus tracking and multicolor imaging, revealing ADD1, acting as a molecular switch, mediating actin branch dynamics with broad relevance to the transport of endocytic viruses or cargoes. Furthermore, we demonstrated that ADD1 downregulation impaired IAV infection in vivo. These data suggested that ADD1 might represent a promising target for anti‐influenza drug discovery.

## 4 Experimental Section

### Cell Culture and Virus

Human embryonic kidney 293T (HEK293T), adenocarcinomic human alveolar basal epithelial (A549), Porcine Kidney‐15 (PK‐15), and Madin‐Darby canine kidney (MDCK) cells were purchased from the American Type Culture Collection (ATCC, USA). HEK293T, A549, MDCK, and PK‐15 cells were cultured in RPMI 1640 (SH30809.01, HyClone, USA), Ham's/F‐12 medium (SH30026.01, HyClone, USA), and Dulbecco's Modified Eagle's medium (DMEM) (SH30243.01, HyClone, USA), respectively. All media were supplemented with 10% fetal bovine serum (FSP500, ExCell, China), and the cells were incubated at 37 °C with 5% CO_2_ for optimal growth conditions.

A/swine/Hubei/221/2016 (HuB/H1N1), A/swine/Henan/F26/2017 (F26/H1N1), A/Puerto Rico/8‐SV14/1934 (PR8/H1N1), A/Hunan/42443/2015 (HuN/H1N1), A/Chicken/Hubei/115/2016 (115/H9N2), and PEDV were preserved in the laboratory. The recombinant vesicular stomatitis virus encoding green fluorescence protein (VSV‐GFP) was kindly provided by Harbin Veterinary Research Institute. The recombinant pseudorabies virus encoding green fluorescence protein (PRV‐GFP) was kindly provided by Professor Zhengfei Liu at Huazhong Agricultural University.

### Antibodies

The antibodies used in this study include the following: Rabbit polyclonal anti‐M1 (GTX125928, GeneTex, USA), Rabbit polyclonal anti‐M1 (ab22396, Abcam, UK), Rabbit polyclonal anti‐ARPC5 (16717‐1‐AP, Proteintech, China), Rabbit polyclonal anti‐NP (GTX125989, GeneTex, USA), Rabbit polyclonal anti‐HA (GTX127357, GeneTex, USA), Rabbit polyclonal anti‐ADD1 (10791‐1‐AP, Proteintech, China), Mouse monoclonal anti‐DYKDDDDK (66008‐4‐Ig, Proteintech, China), Mouse monoclonal anti‐HA (66006‐2‐Ig, Proteintech, China), Mouse monoclonal anti‐GAPDH (60004‐1‐Ig, Proteintech, China), Mouse monoclonal anti‐RhoA (66733‐1‐Ig, Proteintech, China), Rabbit polyclonal anti‐RhoB (14326‐1‐AP, Proteintech, China), Rabbit polyclonal anti‐ARPC1B (28368‐1‐AP, Proteintech, China), Rabbit polyclonal anti‐myosin IIB (21403‐1‐AP, Proteintech, China), Rabbit polyclonal anti‐Lamin A/C (10298‐1‐AP, Proteintech, China), Rabbit monoclonal anti‐CDC42 (A5689, Selleck, China), Rabbit monoclonal anti‐RAB5 (3547, Cell Signaling Technology, USA), Rabbit polyclonal anti‐Phospho‐ADD1 (Ser726) (11182, Signalway Antibody, USA), Rabbit polyclonal anti‐Phospho‐MRLC1 (Thr19+Ser20) (AF8010, Affinity, AU), Rabbit polyclonal anti‐Phospho‐EGFR (Tyr1068) (AP0301, ABclonal, China), Rabbit polyclonal anti‐Clathrin heavy chain (A12423, ABclonal, China), Cy3 goat anti‐mouse IgG (H+L) (AS008, ABclonal, China), Cy3 goat anti‐rabbit IgG (H+L) (AS007, ABclonal, China), FITC goat anti‐mouse IgG (H+L) (AS001, ABclonal, China), Goat polyclonal anti‐mouse IgG‐HRP (Cat# BF03001, Biodragon, China), and Goat polyclonal anti‐rabbit IgG‐HRP (Cat# BF03008, Biodragon, China).

### Generation of Target Gene Knockout and Stably Overexpression Cell Lines

The ADD1 knockout PK‐15 cells were generated using the CRISPR/Cas9 system. Briefly, the paired ADD1 sgRNA was cloned into the lentiCRISPR‐v2 vector. The recombinant vector was co‐transfected with pMD2.G and psPAX2 at a ratio of 20:15:6 into HEK293T cells to generate the lentivirus. PK‐15 cells were transduced with the harvested lentivirus and subjected to selection for positive polyclonal clones using puromycin (S4717, Selleck, USA), followed by being seeded in 96‐well plates to obtain monoclonal cells by limited dilution. The knockout efficiency of ADD1 was determined by immunoblotting and Sanger sequencing (Tsingke, China).

GFP‐tagged RAB5A, RAB7A, and ARPC5 sequences were amplified from the cDNA library of PK‐15 cells. The above amplified fragments and GFP‐tagged lifeact were cloned into the pLenti‐puro vector. Subsequently, these constructs were co‐transfected with pMD2.G and psPAX2 into HEK293T cells to generate lentiviruses. Finally, WT or ADD1‐KO PK‐15 cells were transduced with the harvested lentivirus, and positive clones were sorted by FACS and obtained by limited dilution.

### Cell Viability Assay

Cell viability was assessed according to the instructions of the Cell Counting Kit‐8 (CCK‐8) (GK10001, GLPBIO, USA). Briefly, WT or ADD1‐KO cells were seeded in 96‐well plates. Cell viability was measured at 12, 24, and 36 h post‐seeding. 10 µl CCK‐8 reagent was added to the cells and incubated at 37 °C in the dark for 1 h. The OD values at 450 nm were measured using a microplate reader.

### Viral Infection and Titration

PK‐15 cells were washed twice with serum‐free medium and then infected with HuB/H1N1 (MOI = 0.01) at 37 °C. After 1 h of incubation, the cells were washed twice with serum‐free medium, followed by incubation with DMEM medium supplemented with 0.1 µg mL^−1^ Trypsin‐TPCK (T1426, Sigma, USA) at 37 °C. Subsequently, the cell supernatants were harvested at indicated timepoints and titrated on MDCK cells. Briefly, the cell supernatants were serially diluted and incubated with MDCK cells at 37 °C for 1 h. The inoculum was then replaced with DMEM medium containing 0.25 µg mL^−1^ Trypsin‐TPCK at 37 °C for 72 h. Virus titers were determined using the Reed‐Muench method, as described previously.^[^ ^60^ ^]^

### Binding Assay

For flow cytometry experiments, WT or ADD1‐KO PK‐15 cells were infected with HuB/H1N1 (MOI = 50) at 4 °C to allow virus binding, but not internalization. After 1 h incubation, cells were washed 5 times with cold PBS to remove the unbound virus, followed by digestion with trypsin. The digested cells were fixed with 4% paraformaldehyde (PFA) for 10 min at room temperature (RT), and then rewashed and blocked with 1% BSA in PBS. The cells were then washed and stained with anti‐HA mAb (produced by the laboratory) and corresponding FITC goat anti‐mouse IgG (H+L) (AS001, ABclonal, China). Samples were analyzed by flow cytometry.

For indirect immunofluorescence experiments, WT or ADD1‐KO PK‐15 cells were infected with HuB/H1N1 (MOI = 50) at 4 °C for 1 h. After washing 5 times with cold PBS, cells were fixed with 4% PFA for 10 min at RT, then blocked with 1% BSA in PBS. The cells were stained with primary antibodies and a fluorescent secondary antibody, as described above, and then stained with DAPI (C1002, Beyotime, China) for 10 min at RT. Samples were analyzed using a confocal microscope (A1 HD25, Nikon, Japan).

### Internalization Assay

WT or ADD1‐KO PK‐15 cells were infected with HuB/H1N1 (MOI = 50) at 4 °C for 1 h and then shifted to 37 °C for 0.5 h to allow virus internalization. The cells were fixed with 4% PFA for 10 min and then treated with 0.2% Triton X‐100 for 10 min, followed by blocking with 1% BSA in PBS. The cells were stained with primary antibodies and a fluorescent secondary antibody, as described above. The internalized viral HA was analyzed using a confocal microscope, and cell boundaries were judged by Differential Interference Contrast (DIC) (A1 HD25, Nikon, Japan).

### Acid Bypass Assay

Acid bypass assay was performed as described previously.^[^ ^28^, ^61^ ^]^ WT or ADD1‐KO PK‐15 cells were infected with HuB/H1N1 (MOI = 10) at 4 °C for 1 h. After being washed twice with cold DMEM, the cells were then incubated with warm medium (DMEM supplemented with either 50 mM HEPES (pH 7.4) or 50 mM citric acid (pH 5.0)) for 2 min at 37 °C. Next, cells were immediately washed 2 times with cold DMEM and incubated with warm medium (DMEM supplemented with 50 mM HEPES, pH 7.4). At 6 hpi, cells were collected for immunoblotting analysis of NP expression using a rabbit anti‐NP pAb (GTX125989, GeneTex, USA).

### Acid Bypass M1 Uncoating Assay

To detect M1 uncoating by inducing viral fusion at the plasma membrane, an acid bypass uncoating assay was performed as described previously.^[^ ^61^, ^62^ ^]^ WT or ADD1‐KO PK‐15 cells were infected with HuB/H1N1 (MOI = 50) at 4 °C for 1 h. After being washed twice with cold DMEM, the cells were then incubated with warm medium (DMEM supplemented with either 50 mM HEPES (pH 7.4) or 50 mM citric acid (pH 5.0)) for 2 min at 37 °C. Next, cells were immediately washed 2 times with cold DMEM and incubated with warm medium (DMEM supplemented with 50 mM HEPES, 1 mM cycloheximide, 20 mM NH_4_Cl, pH 7.4) to block endosome acidification. Cells were further incubated at 37 °C for 15 min, after which the cells were fixed and processed for indirect immunofluorescence (IIF).

### Synchronized Penetration Assay at Late Endosomes (NH_4_Cl Washout)

PK‐15 cells expressing GFP‐RAB7, grown on confocal dishes (BS‐20‐GJM, Biosharp, China), were infected with HuB/H1N1 (MOI = 50) at 4 °C for 1 h. Subsequently, the cells were washed and warmed to 37 °C for 0.5 h to allow virus endocytosis in the presence of 1 mM cycloheximide, after which the medium was replaced with STOP medium (DMEM, 50 mM HEPES, pH adjusted to 7.4 and supplemented with 20 mM NH_4_Cl immediately before use) in the presence of 1 mM cycloheximide and incubated for 1.5 h to allow virus particles to accumulate in the late endosomes. The STOP medium was subsequently removed to enable endosomal re‐acidification; meanwhile, the cells were treated with CK‐636 (100 µM), EG‐011 (50 nM), or bafilomycin A1 (100 nM) in the presence of cycloheximide for 0.5 h, after which the cells were fixed and processed for indirect immunofluorescence (IIF).^[^ ^62^ ^]^

### vRNP Nuclear Import Assays

WT or ADD1‐KO PK‐15 cells were infected with HuB/H1N1 (MOI = 20) at 4 °C for 1 h. Subsequently, the cells were washed and warmed to 37 °C for 3 h to allow virus nuclear import in the presence or absence of 1 mM cycloheximide, after which the cells were fixed and processed for indirect immunofluorescence (IIF).

### Virus Labeling

Viruses were labeled with QDs as previously described.^[^ ^15^ ^]^ Briefly, HuB/H1N1 was incubated with 0.5 mg Sulfo‐NHS‐LC‐Biotin (A39257, Thermo, USA) on ice for 4 h. The unbound biotin was removed by ultrafiltration centrifugal tube (50 mL/30 kDa), and the biotinylated HuB/H1N1 (MOI = 50) was collected to infect the PK‐15 cells on ice for 30 min. After washing twice with cold DMEM, cells were incubated with 3 nM streptavidin‐modified QDs (SA‐QDs) (QS605, Wuhan Jiayuan Quantum Dots Co., Ltd, China) on ice for 30 min to label biotinylated HuB/H1N1. Subsequently, cells were washed with cold DMEM to remove redundant QDs and then warmed to 37 °C to track the movement of QDs‐labeled viruses using a confocal microscope (A1 HD25, Nikon, Japan). Images were acquired every 5 s for a duration of 5 min.

Viruses were labeled with 3,3′dioctadecyloxacarbocyanine (DiOC18) and octadecyl rhodamine B (R18) as described previously.^[^ ^29^ ^]^ Briefly, DiOC18 (D275, Thermo, USA) and R18 (O246, Thermo, USA) were mixed at concentrations of 33 µM and 67 µM to form the probe mixture. Next, 6 µl of the mixture was incubated with HuB/H1N1 (MOI = 150, 1 mL) overnight. The redundant DiOC18 and R18 were removed by a 0.22 µm pore size filter (BS‐PES‐22, biosharp, China). Subsequently, the dual‐labeled HuB/H1N1 (MOI = 50) was utilized to infect WT or ADD1‐KO PK‐15 cells. Cells were fixed at 0 or 1.5 hpi to detect membrane fusion using a confocal microscope (A1 HD25, Nikon, Japan).

### High‐Throughput Microscopy and Image Analysis

PK‐15 cells expressing GFP‐lifeact were seeded in 96‐well plates and then infected with or without R18‐labeled HuB/H1N1 (MOI = 50) on ice for 1 h. After washing twice with cold DMEM, the cells were loaded into a high‐content imaging system (Opera Phenix, PE, USA) to track the dynamic rearrangement of F‐actin. Images were acquired every 3 min for a duration of 2.5 h (37 °C, 5% CO_2_).

### Transmission Electron Microscope

WT or ADD1‐KO PK‐15 cells were seeded in 100 mm dishes and then infected with HuB/H1N1 (MOI = 50) on ice for 1 h (0 hpi). After washing twice with cold DMEM, some cells were fixed with 2.5% glutaraldehyde (0 hpi), and some were shifted to 37 °C for 1 h incubation (1 hpi) and then fixed as described above. The fixed cells were collected using a cell scraper to detect the intracellular location of virus by transmission electron microscope (Tecnai G2 20 TWIN, FEI, USA).

### Scanning Electron Microscope

The cytoskeletal components were observed using scanning electron microscopy, following a previously established protocol.^[^ ^63^ ^]^ WT or ADD1‐KO PK‐15 cells were seeded on 20 mm round coverslips to reach 30–40% confluency, and the cells were rinsed with warm PBS and incubated with an extraction solution (1% Triton X‐100, 2% PEG (MW 20,000‐40,000), 5 µM phalloidin, and 5 µM paclitaxel in PEM buffer) for 5 min at RT. Afterward, the cells were rinsed with PEM buffer (100 mM PIPES, 1 mM MgCl_2_, 1 mM EGTA, pH 6.9) for a few seconds each time and underwent incubation with a glutaraldehyde solution (2% glutaraldehyde, 0.1 M sodium cacodylate, pH 7.3) for 1 h at RT. The glutaraldehyde solution was then replaced with tannic acid (0.1% tannic acid in distilled water) for 20 min at RT. Subsequently, the cells were rinsed three times with distilled water, and 0.2% uranyl acetate in distilled water was added and incubated for 20 min at RT. Following a 10‐min rinse with distilled water each time, the cells underwent ethanol dehydration, critical point drying (CPD), and platinum and carbon coating. The samples were then analyzed using a scanning electron microscope (SU8010, Hitachi, Japan).

### GST Pulldown Assays and Co‐Immunoprecipitation (Co‐IP)

The Glutathione S‐transferase (GST)‐fused Rhotekin‐RBD, Diaph1‐RBD, PAK1‐PBD, Rabaptin5‐R5BD or GST proteins were expressed from *Escherichia coli* BL21 (TSC‐E01, TSINGKE, China) and incubated with GST Resin (P2020, Solarbio, China) at 4 °C for 4 h to form beads‐GST recombinant protein complexes. After washing with ice‐cold IP lysis buffer (P0013, Beyotime, China), the beads‐GST recombinant protein complexes were incubated with the lysates of WT or ADD1‐KO PK‐15 cells at 4 °C for 6 h. Subsequently, the precipitated complexes were washed 5 times with ice‐cold IP lysis buffer, followed by immunoblot analysis with the indicated antibodies.

For co‐immunoprecipitation, HEK293T cells were transfected with the aforementioned plasmids using Lipofectamine 8000 (C0533, Beyotime, China). After 24 h of transfection, cells were collected and lysed with IP lysis buffer (supplemented with a protease inhibitor cocktail) on ice for 30 min. Subsequently, the lysates were incubated with bead‐antibody complexes at 4 °C for 8 h to precipitate the targeted proteins. After washing and diluting with ice‐cold IP lysis buffer, the samples were separated on SDS‐PAGE. Next, the samples were transferred to nitrocellulose for immunoblotting, and the images were obtained using the ECL detection system (Tanon 5200, Tanon, China).

### Mouse Models

Animal experiments were approved by the Animal Management and Ethics Committee of Huazhong Agricultural University (No. HZAUMO‐2023‐0316 and HZAUMO‐2023‐0326).

For EG‐011 treatment in vivo, 4‐6‐week‐old female BALB/c mice were randomly divided into 3 groups: PBS control group, solvent treated group, and EG‐011 treated group. The mice were intraperitoneally injected with EG‐011 (dissolved in a solvent containing 10% DMSO, 40% PEG300, 5% Tween‐80, and 45% saline, at the concentration of 10 mg kg^−1^) or an equal volume of vehicle (the solvent) for 6 days, and challenged with 30 pfu of HuN/H1N1 or mock‐treated on day 0. The weight loss and survival rate of the mice were monitored daily for 14 days, and the lungs of each group (n = 3) harvested on days 3 and 5 post‐challenge were either homogenized to evaluate the viral titers or fixed in 4% formaldehyde for H&E staining and immunofluorescence staining for histopathological analysis.

For siRNA treatment in vivo, 4‐6‐week‐old female BALB/c mice were randomly divided into 3 groups: PBS control group, si‐control treated group, and si‐ADD1 treated group. The mice were nasally instilled with 50 µl of PBS, 5 nmol si‐control, or 5 nmol si‐ADD1 on day ‐1 and day 1 separately, and challenged with 30 pfu of HuN/H1N1 or mock‐treated on day 0. The weight loss and survival rate of mice were monitored daily for 14 days. On days 3 and 5 post‐challenge, the mice of each group (n = 3) were anesthetized and sacrificed, and the lungs were either homogenized or fixed in 4% formaldehyde. The supernatants of homogenized lungs were used for detecting ADD1 expression or assessing the viral titers. The fixed lung tissues were used for H&E staining and immunofluorescence staining for histopathological analysis. Cholesterol‐conjugated and 2′‐ OME modified ADD1‐siRNA (si‐ADD1, targeted sequence 5′‐ GGUCCCAGCUUAUCUACAATT‐3′) was synthesized by GenePharma.

### Image Analysis

The signals of viruses and motor proteins were tracked using Imaging‐Pro Plus to acquire the moving distance. The accumulated distance vs. time or instantaneous velocity vs. time plots were analyzed by GraphPad Prism v.8.0. Each frame of the video stack was processed by deconvolution.

### Statistical Analysis

All statistical analyses were performed using GraphPad Prism v.8.0. The results are shown as the mean ± standard deviation of the triplicate determinations. Statistical significance was determined by a two‐tailed Student's t‐test (ns, *P *> 0.05; *P < 0.05; **P < 0.01; ***P < 0.001; ****P < 0.0001).

## Conflict of Interest

The authors declare no conflict of interest.

## Supporting information



Supporting Information

Supplemental Video 1

Supplemental Video 2

Supplemental Video 3

Supplemental Video 4

Supplemental Video 5

Supplemental Video 6

Supplemental Video 7

Supplemental Video 8

Supplemental Video 9

Supplemental Video 10

Supplemental Video 11

Supplemental Table 1

## Data Availability

The data that support the findings of this study are available from the corresponding author upon reasonable request.
