# Supplementary material for: Adducin‐1 Facilitates Influenza Virus Endosomal Trafficking and Uncoating by Regulating Branched Actin Dynamics and Myosin IIB Activity
Source: Adv Sci (Weinh). 2025 Jun 5;12(28):2417318. doi: 10.1002/advs.202417318 (PMC12302527; doi:10.1002/advs.202417318)
Supplement: Supplementary file 1 — Supporting Information [file ADVS-12-2417318-s002.docx]

# Supporting Information

**Adducin-1 Facilitates Influenza Virus Endosomal Trafficking and Uncoating by Regulating Branched Actin Dynamics and Myosin IIB Activity**

*Meijun Jiang, Jiahui Zou, Yaoming Jin, Chenjun Jiang, Shaoyu Tu, Tong Chen, Jinli Guo, Yanqing Cheng, Meilin Jin, Huanchun Chen, Hongbo Zhou^*^*

**
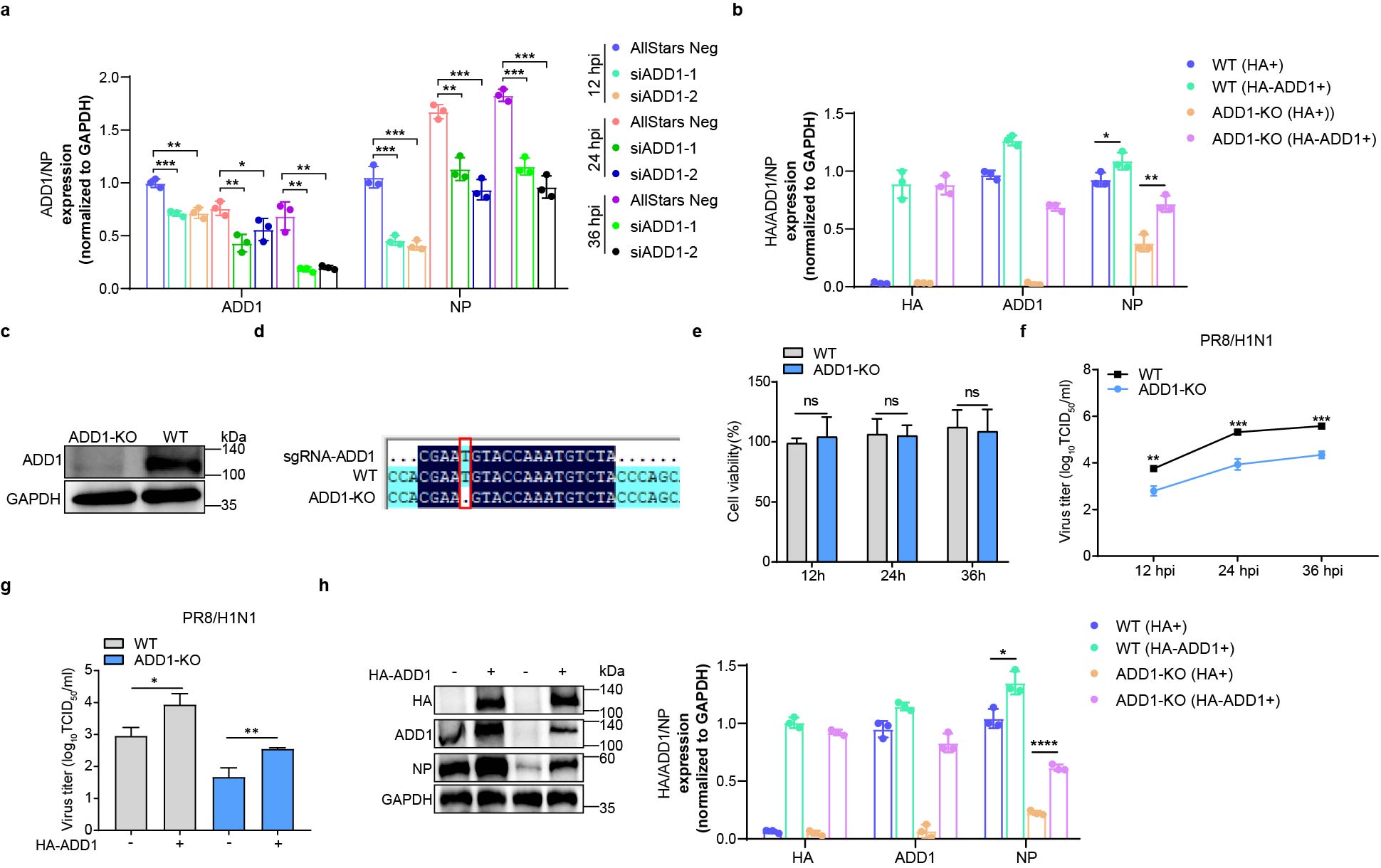
**

**Figure S1.** a) Grayscale analysis quantification of ADD1 and NP protein expression levels in PK-15 cells treated with ADD1 siRNA or negative control siRNA and infected with HuB/H1N1 (*n* = 3). b) Grayscale analysis quantification of HA, ADD1, and NP protein expression levels in WT and ADD1-KO PK-15 cells transfected with the HA vector or HA-ADD1 and infected with HuB/H1N1 (*n* = 3). c, d) The knockout efficiency of ADD1 in A549 cells was measured by Western blot assay (c) and Sanger sequencing (d) (*n* = 3). e) Cell viability of WT and ADD1*-*KO A549 cells was determined by CCK-8 detection (*n* = 8). f) WT and ADD1-KO A549 cells were infected with PR8/H1N1 (MOI = 0.01), and the cell supernatants were subsequently harvested at the indicated times; virus titers were determined by TCID_50_ assay (*n* = 3). g) WT and ADD1*-*KO A549 cells were transfected with HA vector or HA-ADD1 for 24 hours, followed by infection with PR8/H1N1 (MOI = 0.01) to assess viral titers (*n* = 3). h) Immunoblot analysis confirmed HA, ADD1 and NP expression, with grayscale analysis for quantification (*n* = 3). Data are presented as mean ± SD. Statistical analysis was performed using unpaired, two-tailed Student’s t-test. **P* < 0.05; ***P* < 0.01; ****P* < 0.001; *****P* < 0.0001; *ns*, not significant.

**
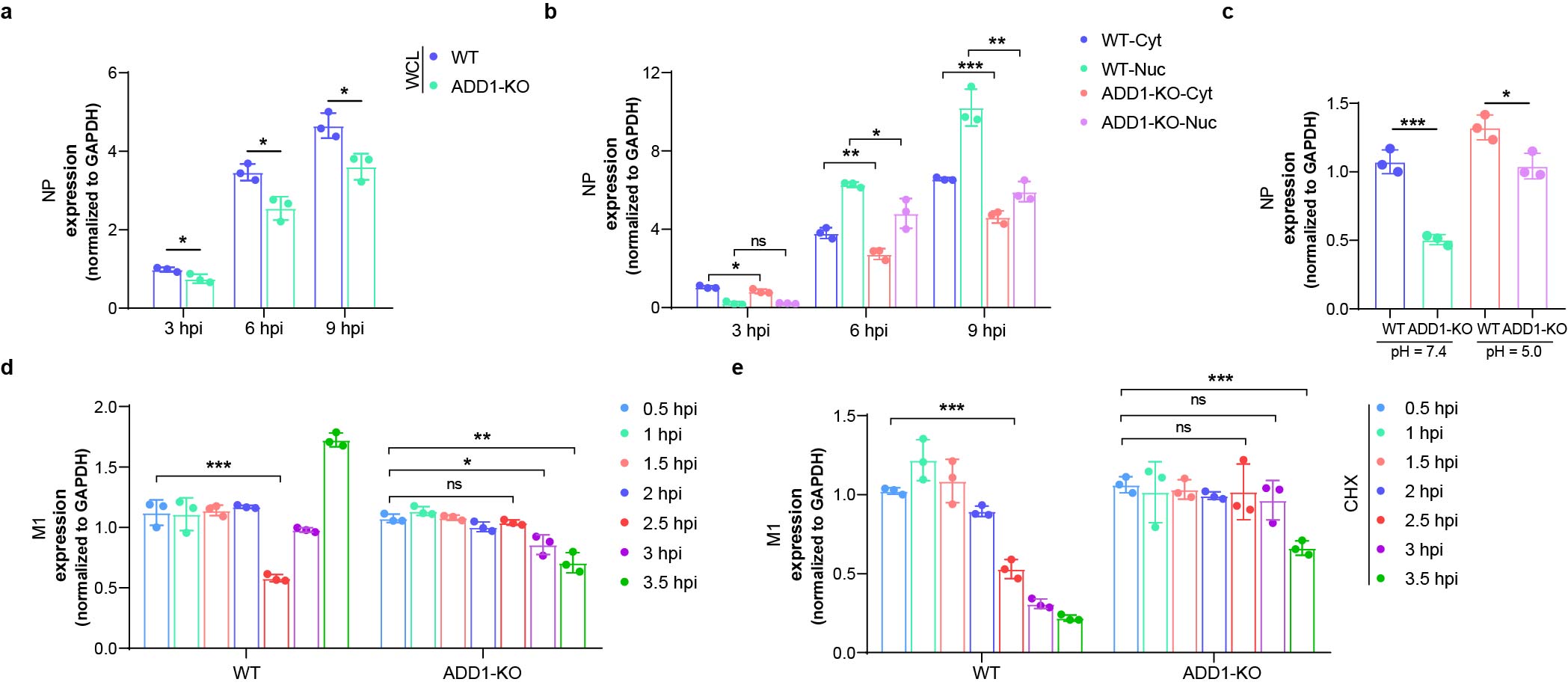
**

**Figure S2.** a) Grayscale analysis quantification of NP protein expression levels in whole cell lysates (WCL) from WT and ADD1-KO PK-15 cells infected with HuB/H1N1 (*n* = 3). b) Grayscale analysis quantification of NP protein expression levels in the cytoplasm and nucleus of WT and ADD1-KO PK-15 cells infected with HuB/H1N1 (*n* = 3). c) Grayscale analysis quantification of NP protein expression levels in WT and ADD1-KO PK-15 cells treated with pH = 7.4 or pH = 4.5 buffer and infected with HuB/H1N1 (*n* = 3). d) Grayscale analysis quantification of M1 protein expression levels in WT and ADD1-KO PK-15 cells infected with HuB/H1N1 (*n* = 3). e) Grayscale analysis quantification of M1 protein expression levels in WT and ADD1-KO PK-15 cells treated with CHX and infected with HuB/H1N1 (*n* = 3). Data are presented as mean ± SD. Statistical analysis was performed using unpaired, two-tailed Student’s t-test. **P* < 0.05; ***P* < 0.01; ****P* < 0.001; *****P* < 0.0001; *ns*, not significant.

**
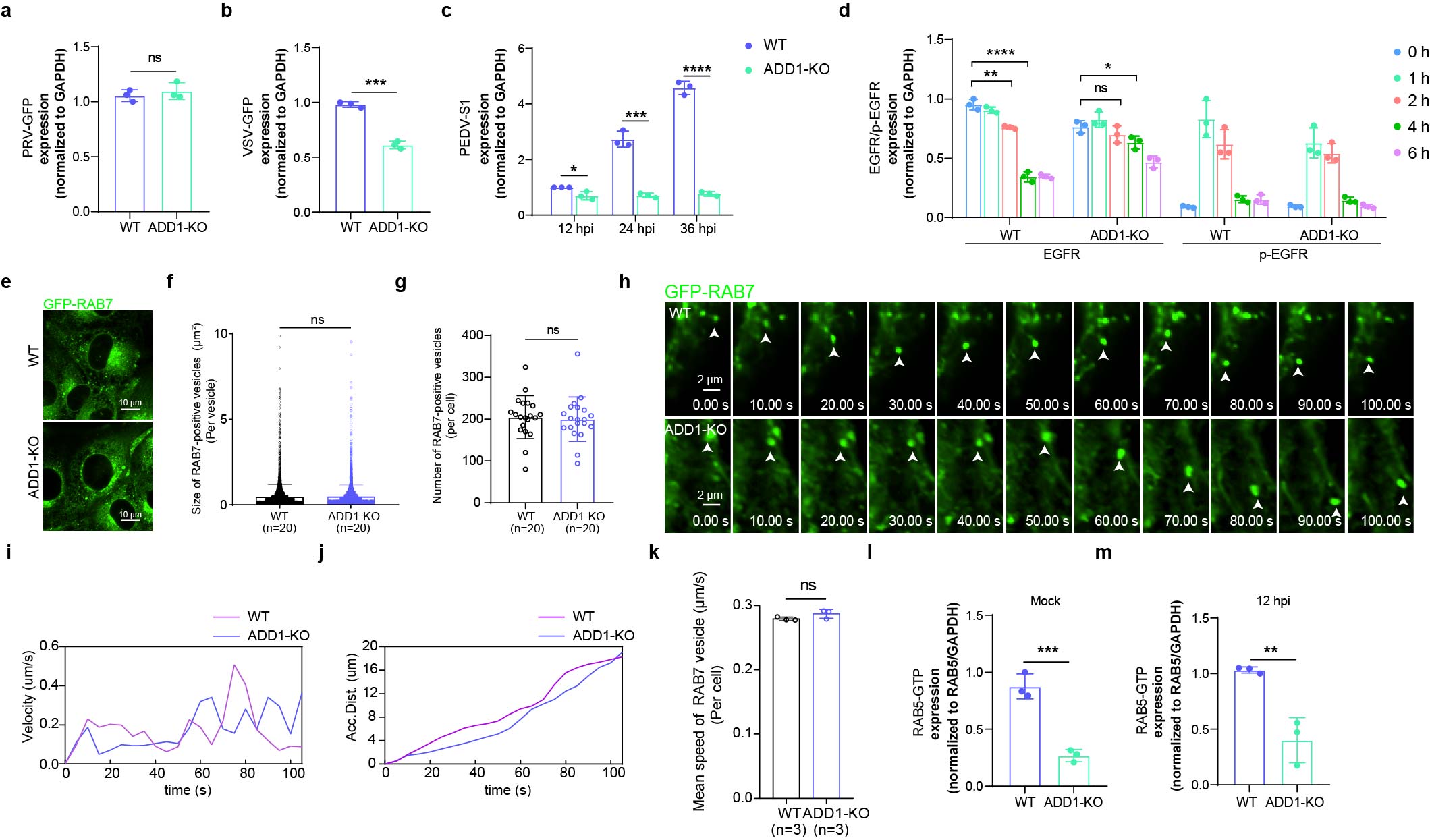
**

**Figure S3.** a) Grayscale analysis quantification of PRV-GFP protein expression levels in WT and ADD1-KO PK-15 cells infected with PRV (*n* = 3). b) Grayscale analysis quantification of VSV-GFP protein expression levels in WT and ADD1-KO PK-15 cells infected with VSV (*n* = 3). c) Grayscale analysis quantification of PEDV-S1 protein expression levels in WT and ADD1-KO PK-15 cells infected with PEDV (*n* = 3). d) Grayscale analysis quantification of EGFR and p-EGFR protein expression levels in WT and ADD1-KO A549 cells treated with EGF (100 ng/mL) (*n* = 3). e-g) WT or ADD1-KO PK-15 cells expressing GFP-RAB7 were subjected to fluorescence imaging (e). The size (f) and the number (g) of RAB7-positive vesicles were quantified, and the values are indicated at the bottom of each image (*n* = 20 cells). Scale bars represent 10 μm. h) Snapshots of the movement of a RAB7-positive vesicle (pointed out with white arrows). i, j) Instantaneous velocity *vs*. time (i), accumulated distance *vs.* time (j) plots of the RAB7-positive vesicle. k) The mean speed of the RAB7-positive vesicle was quantified, and the values are indicated at the bottom of each image (*n* = 3 cells). l) Grayscale analysis quantification of RAB5-GTP protein expression levels in WT and ADD1-KO PK-15 cells (*n* = 3). m) Grayscale analysis quantification of RAB5-GTP protein expression levels in WT and ADD1-KO PK-15 cells infected with HuB/H1N1 (*n* = 3). Data are presented as mean ± SD. Statistical analysis was performed using unpaired, two-tailed Student’s t-test. **P* < 0.05; ***P* < 0.01; ****P* < 0.001; *****P* < 0.0001; *ns*, not significant.


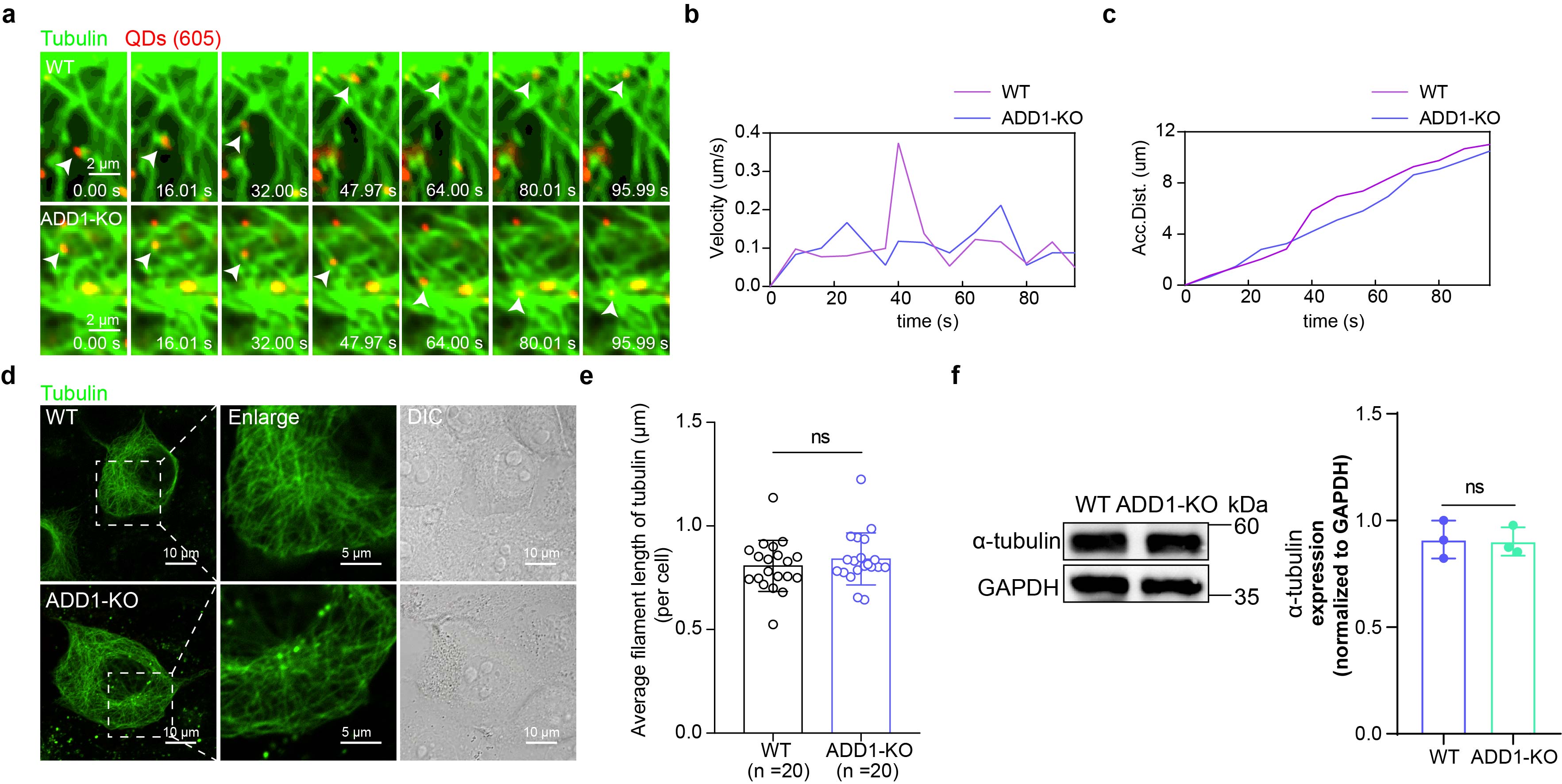


**Figure S4.** a) Snapshots of a virus (pointed out with white arrows) moving on microtubules. b, c) Instantaneous velocity *vs*. time (b), accumulated distance *vs.* time (c) plots of the tracked virus. d, e) WT or ADD1-KO PK-15 cells were stained with TubGreen™ (green) to label tubulin and subjected to fluorescence imaging (d). The length of tubulin (e) was quantified, and the values are indicated at the bottom of each image (*n* = 20 cells). Scale bars represent 10 μm. The scale bars of the enlarged panel represent 5 μm. f) α-tubulin expression in WT or ADD1-KO PK-15 cells was assessed by immunoblot, with grayscale analysis for quantification (*n* = 3). Data are presented as mean ± SD. Statistical analysis was performed using unpaired, two-tailed Student’s t-test. **P* < 0.05; ***P* < 0.01; ****P* < 0.001; *****P* < 0.0001; *ns*, not significant.


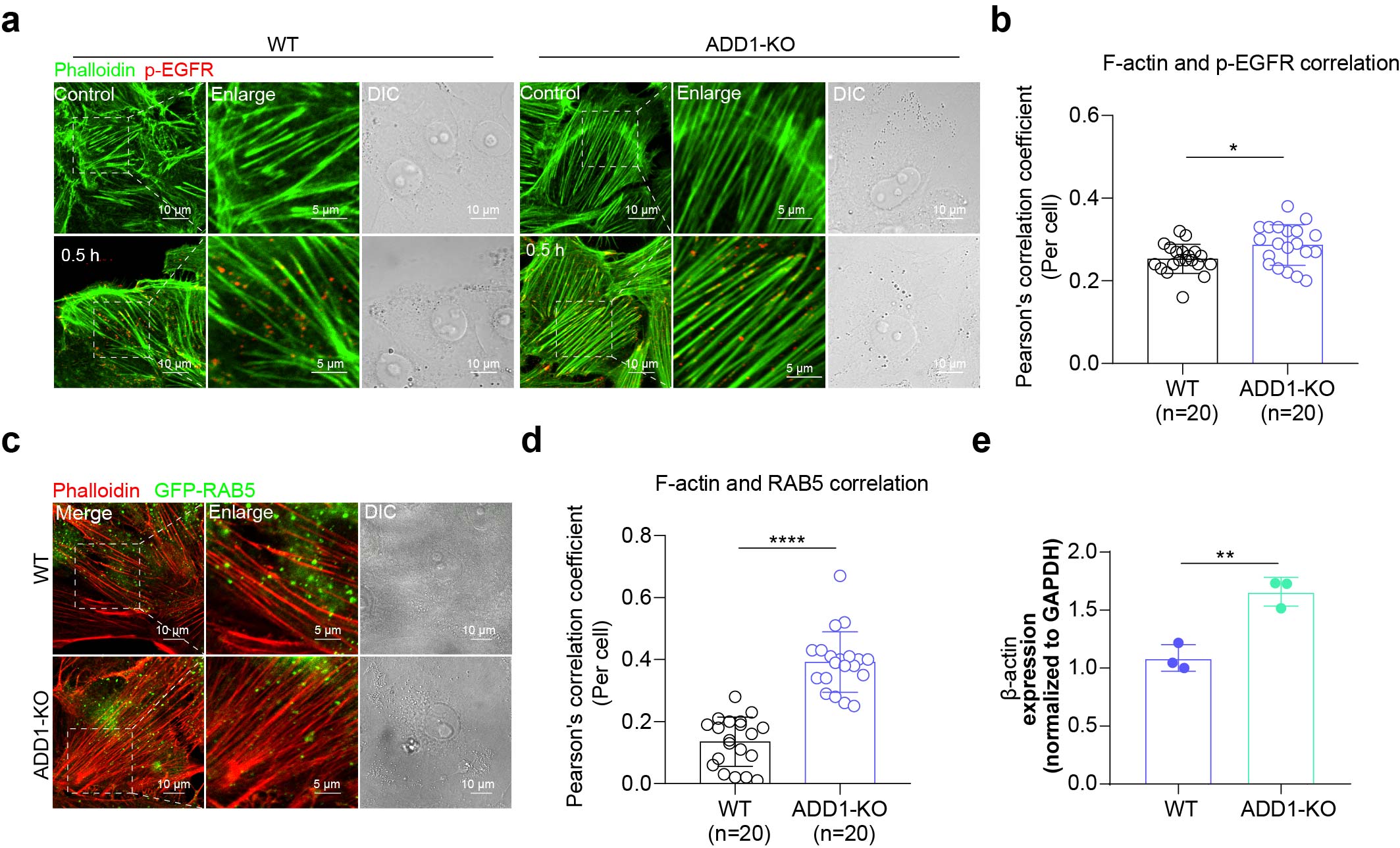


**Figure S5.** a, b) WT or ADD1-KO PK-15 cells were stimulated with EGF (200 ng/mL), and images of the co-localization of p-EGFR (red) with F-actin (green) were acquired using a confocal microscope (a). The Pearson’s correlation coefficient (PCC) of p-EGFR and F-actin was quantified (b). c, d) WT or ADD1-KO PK-15 cells expressing GFP-RAB5 were stained with phalloidin (red) to label F-actin and subjected to fluorescence imaging (c). The Pearson’s correlation coefficient (PCC) of RAB5 and F-actin was quantified (d). The values are indicated at the bottom of each image (*n* = 20 cells). Scale bars represent 10 μm. The scale bars of the enlarged panels represent 5 μm. e) Grayscale analysis quantification of β-actin protein expression levels in WT and ADD1-KO PK-15 cells (*n* = 3). Data are presented as mean ± SD. Statistical analysis was performed using unpaired, two-tailed Student’s t-test. **P* < 0.05; ***P* < 0.01; ****P* < 0.001; *****P* < 0.0001; *ns*, not significant.

**
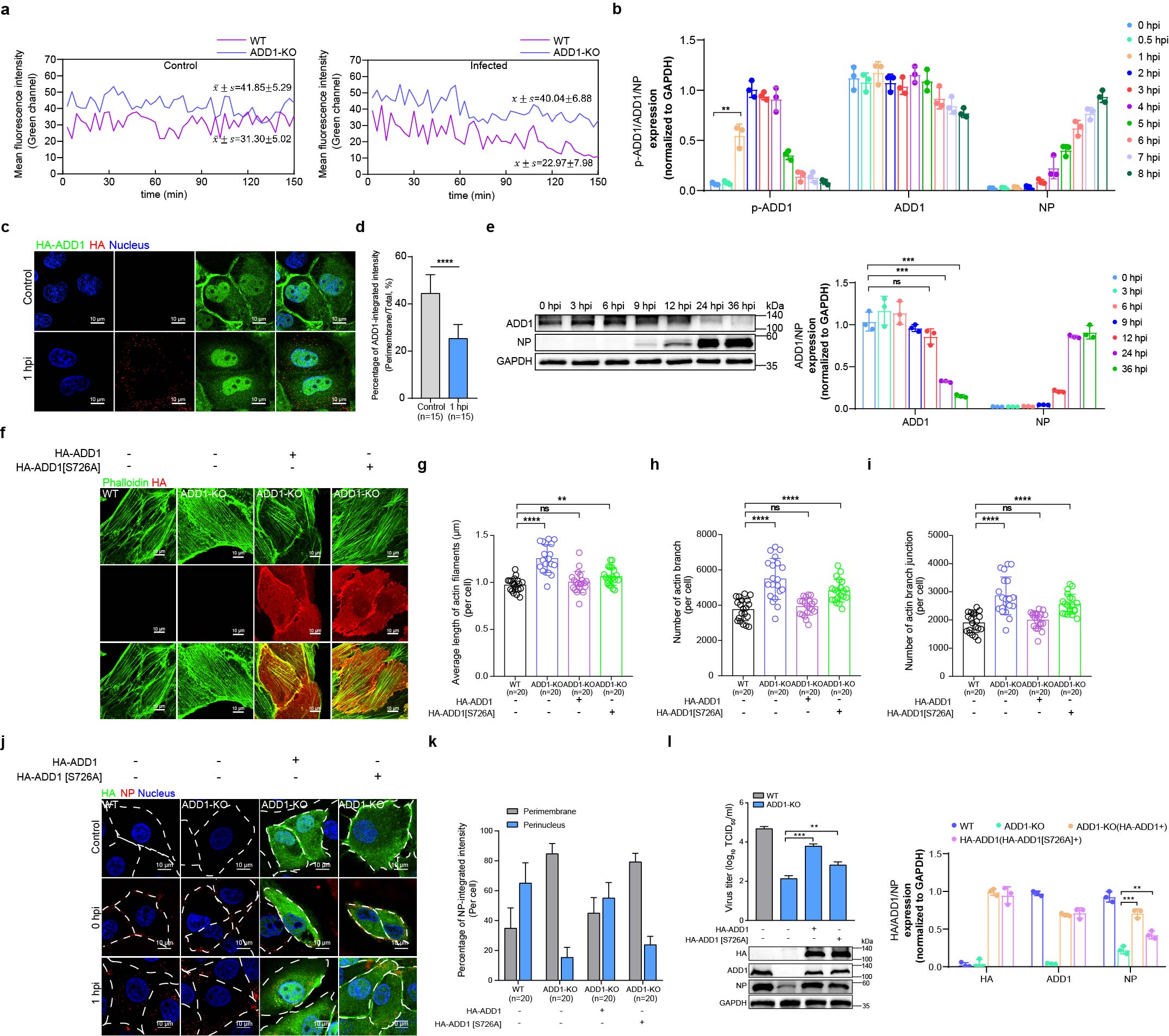
**

**Figure S6.** a) Average fluorescence intensity *vs*. time plots of GFP-lifeact tracked in Videos S9 and S10. b) Grayscale analysis quantification of p-ADD1, ADD1, and NP protein expression levels in WT and ADD1-KO PK-15 cells infected with HuB/H1N1 (*n* = 3). c) HA-ADD1-expressing PK-15 cells were infected with HuB/H1N1 (MOI = 50) or mock-infected, and then immunostained with antibodies against influenza HA (red) and the HA tag (green), followed by fluorescence imaging. d) The perimembrane distribution of HA-ADD1 was analyzed, and the values are indicated at the bottom of each image (*n* = 15 cells). e) WT PK-15 cells were infected with HuB/H1N1 (MOI = 0.01), and the expression levels of ADD1 and NP protein were assessed by immunoblot at the indicated times, with grayscale analysis for quantification (*n* = 3). f-i) HA-ADD1 or HA-ADD1 [S726A] was stably expressed in ADD1-KO cells. The cells were stained with phalloidin (green) to label F-actin and immunostained with antibodies against the HA tag (red), followed by fluorescence imaging (f). The average length of actin filaments (g), the number of actin branches (h), and the number of actin branch junctions (i) were quantified, and the values are indicated at the bottom of each image (*n* = 20 cells). j, k) HA-ADD1 or HA-ADD1 [S726A] was stably expressed in ADD1-KO cells and infected with HuB/H1N1 (MOI = 50) followed by fluorescence imaging (j). The perimembrane or perinuclear distribution of viral NP was analyzed, and the values are indicated at the bottom of each image (*n* = 20 cells) (k). HA (green), NP (red), nucleus (blue). l) HA-ADD1 or HA-ADD1 [S726A] was stably expressed in ADD1-KO cells followed by infection with HuB/H1N1 (MOI = 0.01) to assess viral titers. Immunoblot analysis confirmed HA, ADD1 and NP protein expression, with grayscale analysis for quantification (*n* = 3). Scale bars represent 10 μm. Data are presented as mean ± SD. Statistical analysis was performed using unpaired, two-tailed Student’s t-test. **P* < 0.05; ***P* < 0.01; ****P* < 0.001; *****P* < 0.0001; *ns*, not significant.


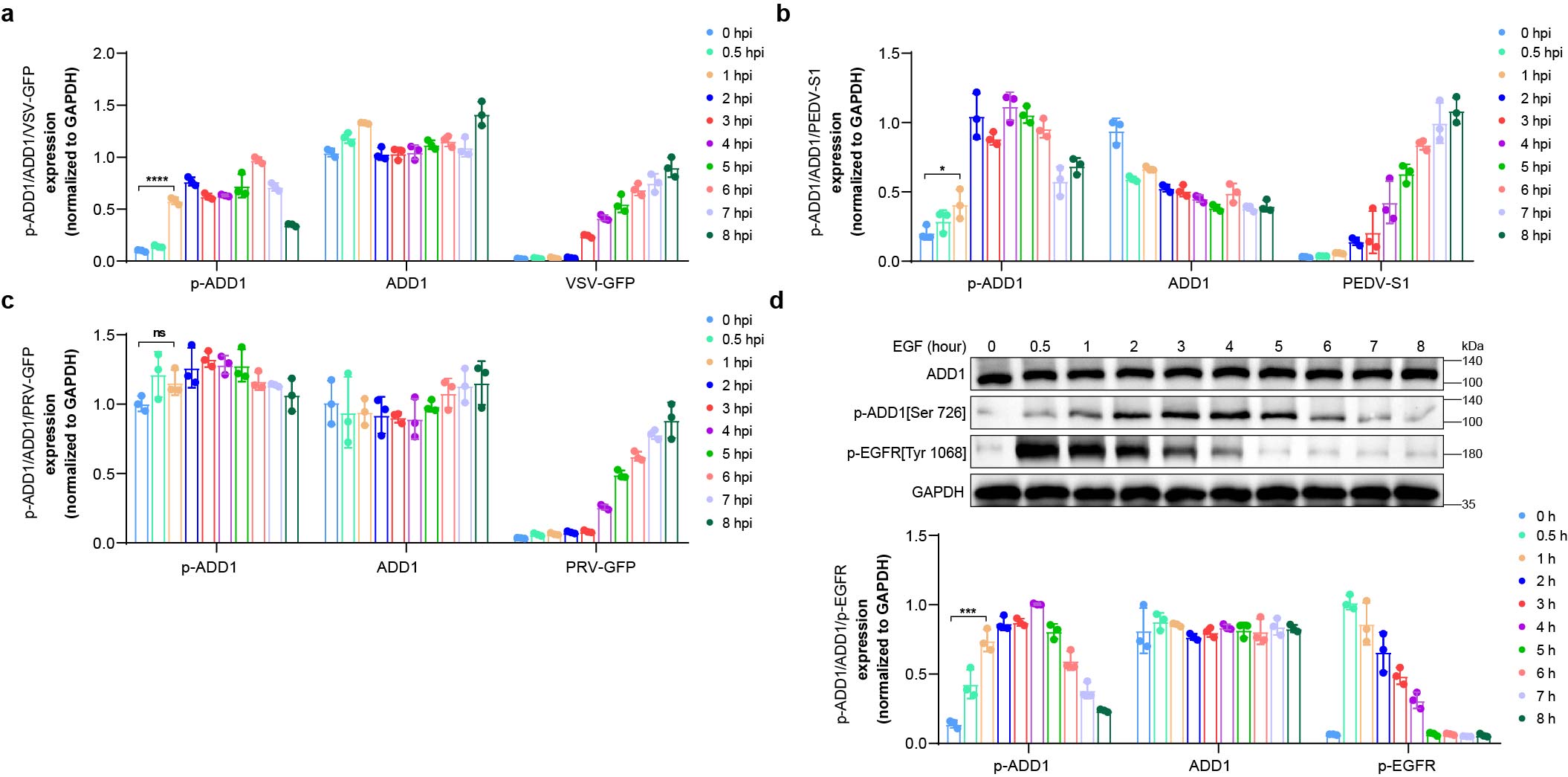


**Figure S7.** a) Grayscale analysis quantification of p-ADD1, ADD1, and VSV-GFP protein expression levels in WT and ADD1-KO PK-15 cells infected with VSV (*n* = 3). b) Grayscale analysis quantification of p-ADD1, ADD1, and PEDV-S1 protein expression levels in WT and ADD1-KO PK-15 cells infected with PEDV (*n* = 3). c) Grayscale analysis quantification of p-ADD1, ADD1, and PRV-GFP protein expression levels in WT and ADD1-KO PK-15 cells infected with PRV (*n* = 3). d) WT PK-15 cells were stimulated with EGF (200 ng), and the expression levels of p-ADD1, ADD1, and p-EGFR protein were assessed by immunoblot, with grayscale analysis for quantification (*n* = 3). Data are presented as mean ± SD. Statistical analysis was performed using unpaired, two-tailed Student’s t-test. **P* < 0.05; ***P* < 0.01; ****P* < 0.001; *****P* < 0.0001; *ns*, not significant.


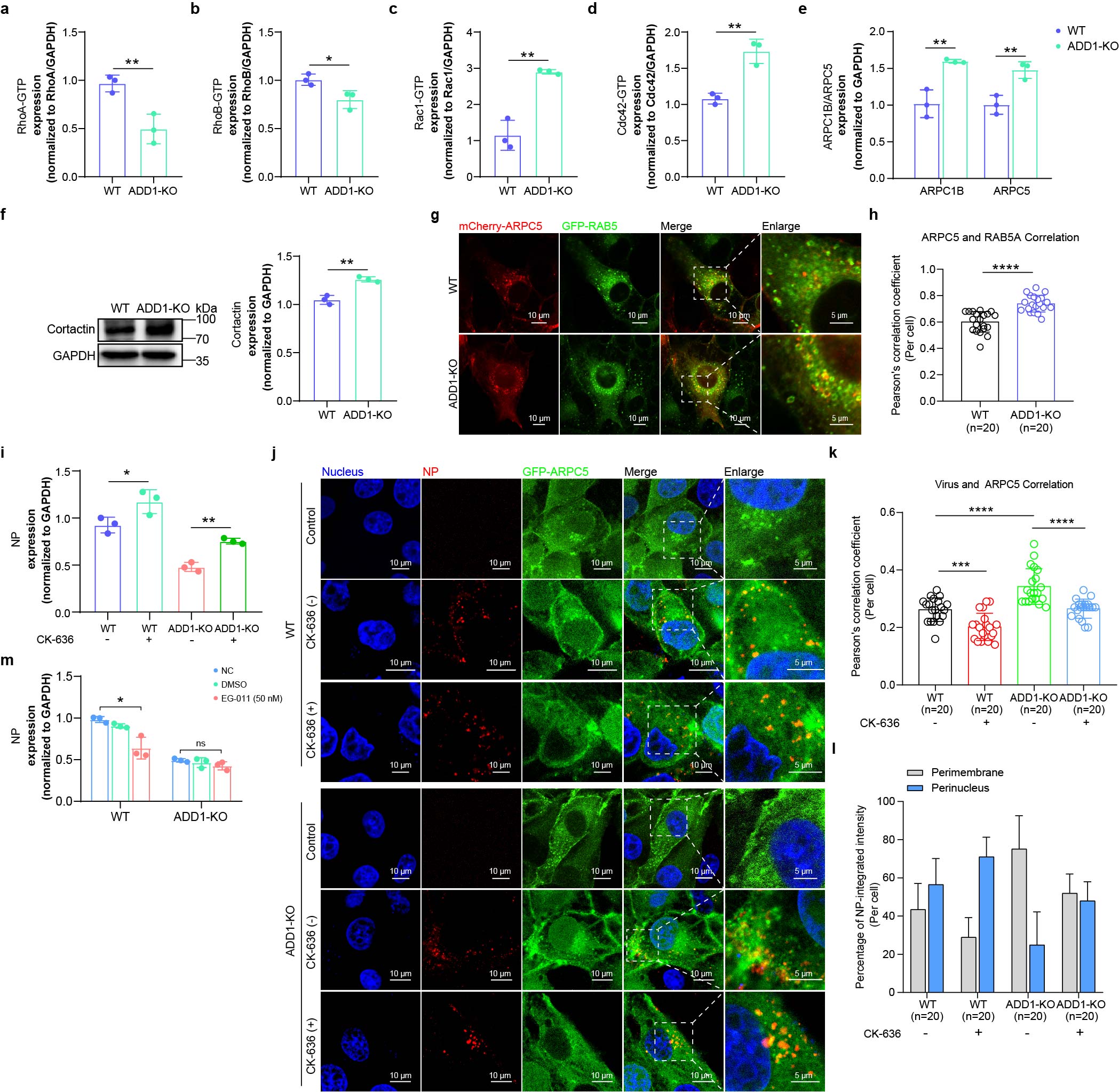


**Figure S8.** a-d) Grayscale analysis quantification of RhoA-GTP (a), RhoB-GTP (b), Rac1-GTP (c), Cdc42-GTP (d) protein expression levels in WT and ADD1-KO PK-15 cells (*n* = 3). e) Grayscale analysis quantification of ARPC1B and ARPC5 protein expression levels in WT and ADD1-KO PK-15 cells (*n* = 3). f) Cortactin expression in WT or ADD1-KO PK-15 cells was assessed by immunoblot, with grayscale analysis for quantification (*n* = 3). g, h) The images showing the co-localization of GFP-RAB5 (green) with mCherry-ARPC5 (red) in WT or ADD1-KO PK-15 cells were acquired using a confocal microscope (g). The Pearson’s correlation coefficient (PCC) of GFP-RAB5 and mCherry-ARPC5 was quantified (h), and the values are indicated at the bottom of each image (*n* = 20 cells). Scale bars represent 10 μm. The scale bars of the enlarged panels represent 5 μm. i) Grayscale analysis quantification of NP protein expression levels in WT and ADD1-KO PK-15 cells treated with or without CK-636 (100 μM) and infected with HuB/H1N1 (*n* = 3). j-l) WT or ADD1*-*KO PK-15 cells were treated with or without CK-636 (100 μM) for 20 minutes after virus endocytosis and subjected to fluorescence imaging (j). The Pearson’s correlation coefficient (PCC) of NP and GFP-ARPC5 was quantified (k), and the values are indicated at the bottom of each image (*n* = 20 cells). The perimembrane or perinuclear distribution of viral NP was analyzed (l), and the values are indicated at the bottom of each image (*n* = 20 cells). Scale bars represent 10 μm. The scale bars of the enlarged panels represent 5 μm. m) Grayscale analysis quantification of NP protein expression levels in WT and ADD1-KO PK-15 cells treated with or without EG-011 (50 nM) and infected with HuB/H1N1 (*n* = 3). Data are presented as mean ± SD. Statistical analysis was performed using unpaired, two-tailed Student’s t-test. **P* < 0.05; ***P* < 0.01; ****P* < 0.001; *****P* < 0.0001; *ns*, not significant.

**
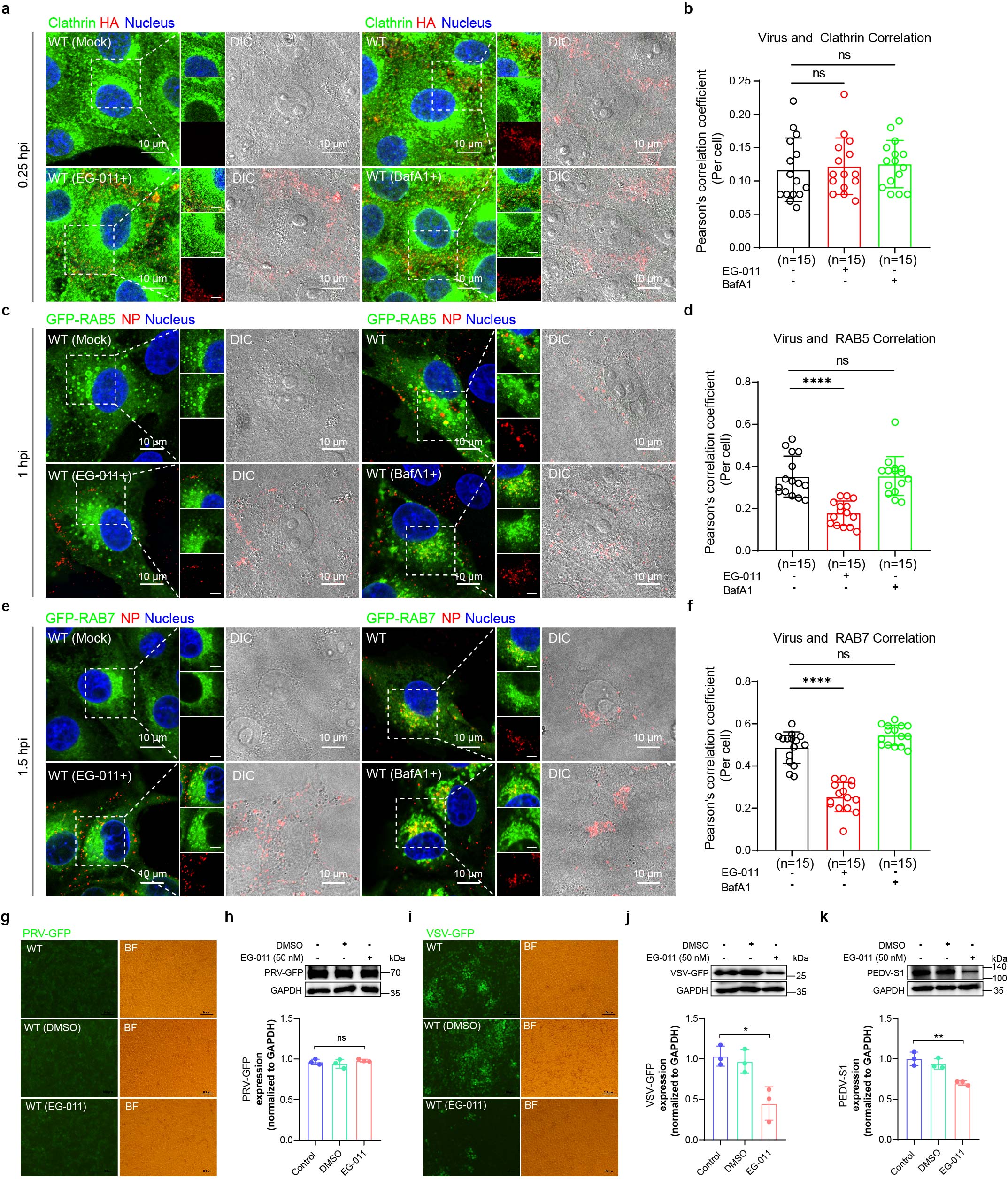
**

**Figure S9.** a-f) WT PK-15 cells were treated with EG-011 (50 nM) for 1 h, and then infected with HuB/H1N1 (MOI = 50). The co-localization of viral HA (red) with Clathrin (green) at 0.25 hpi (a), viral NP (red) with GFP-RAB5 (green) at 1 hpi (c), or viral NP (red) with GFP-RAB7 (green) at 1.5 hpi (e) was acquired using the confocal microscope. The Pearson’s correlation coefficient (PCC) of the virus and Clathrin (b), GFP-RAB5 (d), or GFP-RAB7 (f) was quantified, and the values are indicated at the bottom of each image (*n* = 15 cells). Scale bars represent 10 μm. Scale bars of the enlarged panel represent 5 μm. g-k) WT PK-15 cells were treated with EG-011 (50 nM) for 1 h, and then infected with PRV-GFP (MOI = 0.1) (g, h), VSV-GFP (MOI = 0.1) (i, j), or PEDV (MOI = 0.1) (k). The fluorescence of PRV-GFP or VSV-GFP was visualized by fluorescence microscopy, and the expression levels of GFP (for PRV-GFP and VSV-GFP) or PEDV-S1 protein were determined by immunoblotting, with grayscale analysis for quantification (*n* = 3). Scale bars represent 100 μm. Data are presented as mean ± SD. Statistical analysis was performed using unpaired, two-tailed Student’s t-test. **P*< 0.05; ***P* < 0.01; ****P* < 0.001; *****P* < 0.0001; *ns*, not significant.

**
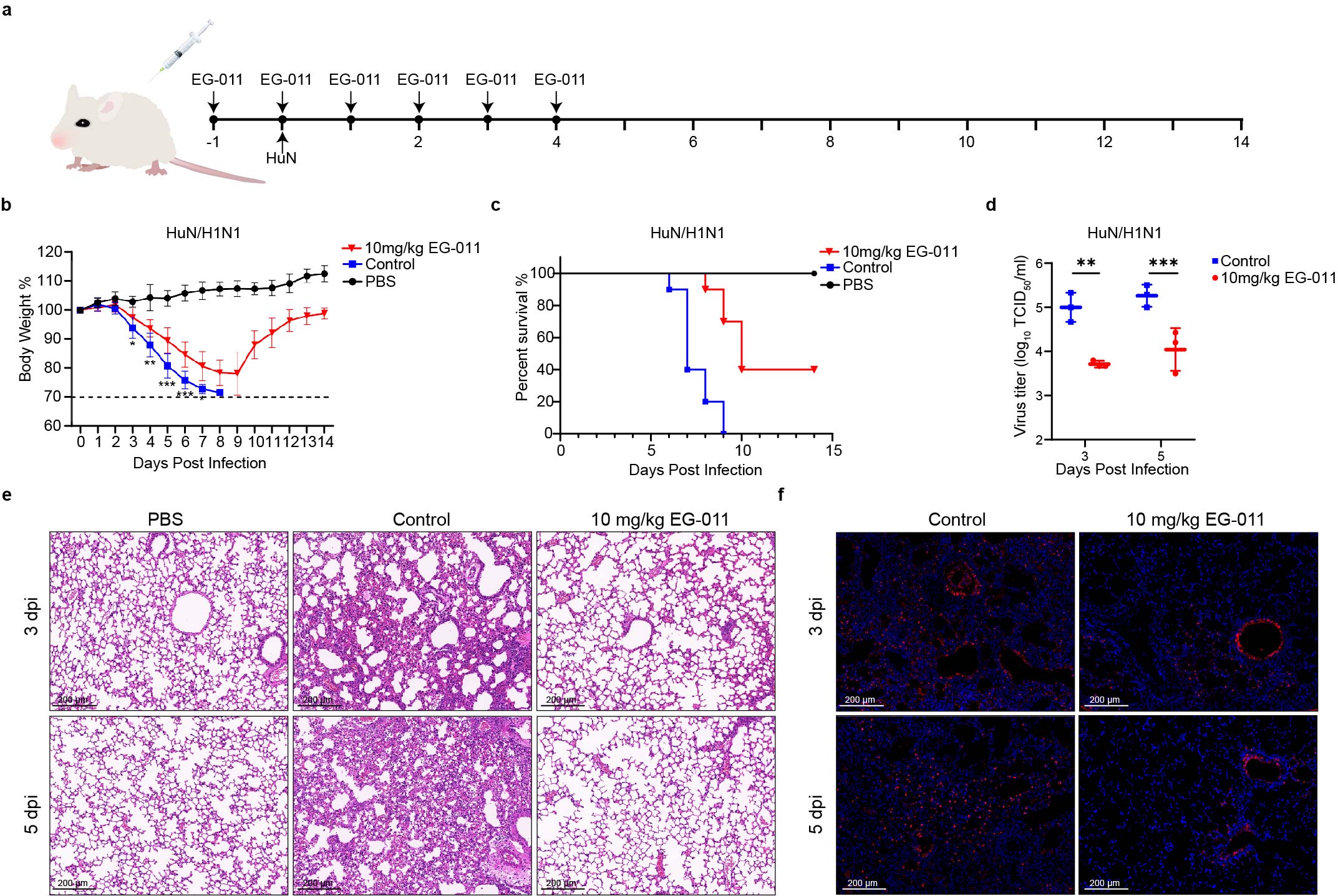
**

**Figure S10.** a) A flowchart of EG-011 treatment and IAV infection in a mouse model. b) Percentage change in body weight of mice was monitored for 14 days. The dotted line represents 70% of initial weight. Data are mean ± SEM (*n* = 10 mice). c) The survival of mice was monitored for 14 days. (*n* = 10 mice, with a log-rank test [Mantel Cox]). d) On 3 and 5 dpi, mice were euthanized to harvest the lungs and determine viral titers. Data are mean ± SD (*n* = 3 mice). e) The lungs of mice treated with EG-011 and the solvent were stained with hematoxylin and eosin (H&E) for histopathological diagnostics. Scale bars represent 200 μm. (*n* = 3 mice). f) Representative immunofluorescence images of viral NP (red) in lung alveolar epithelial cells. Scale bars represent 200 μm. (*n* = 3 mice). **P*< 0.05; ***P* < 0.01; ****P* < 0.001; *****P* < 0.0001; *ns*, not significant.

**
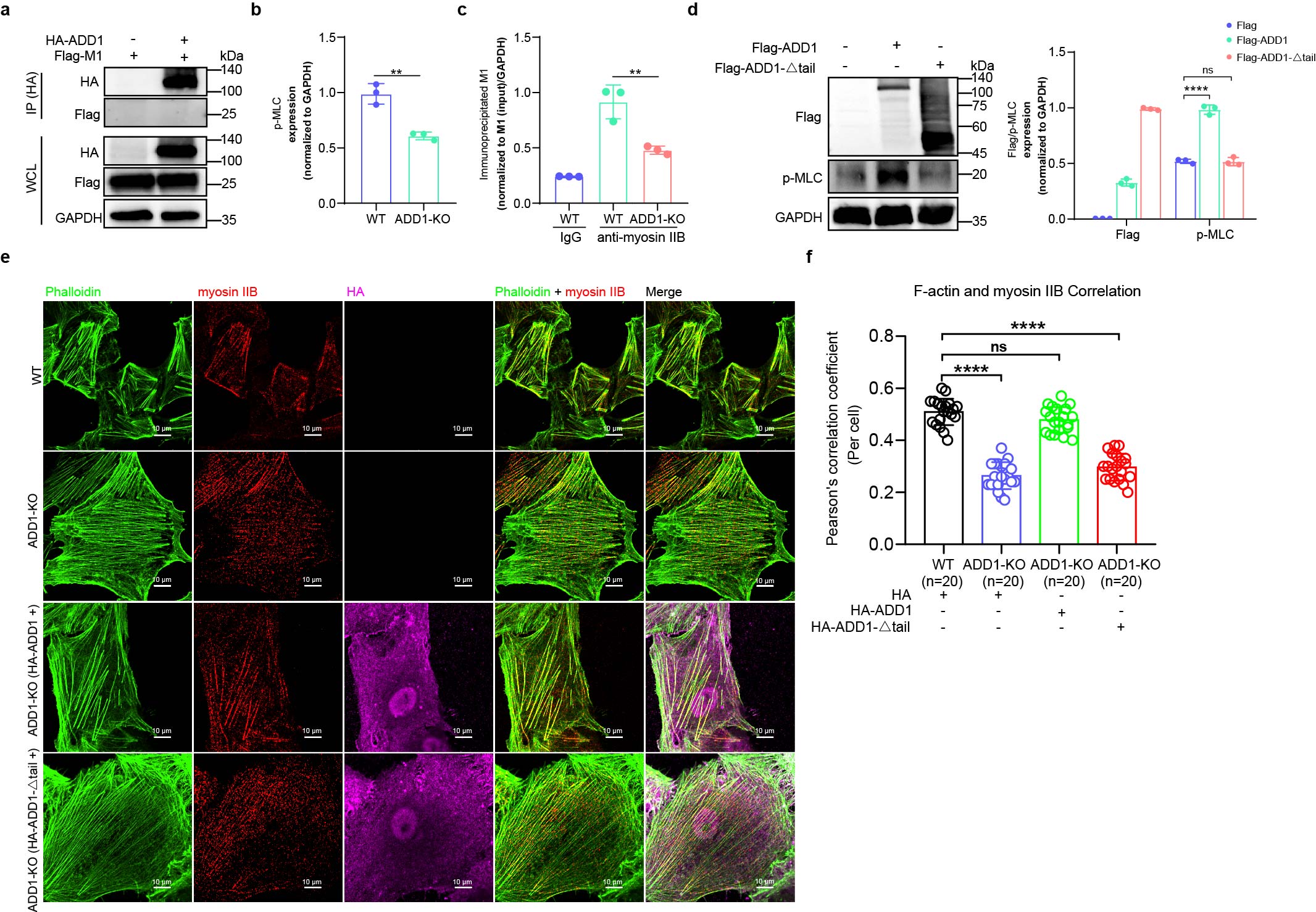
**

**Figure S11.** a) HEK293T cells were transfected with Flag-M1, HA-ADD1, or vector (HA). At 24 hours post-transfection, cells were lysed and subjected to immunoprecipitation assays (*n* = 3). b) Grayscale analysis quantification of p-MLC protein expression levels in WT and ADD1-KO PK-15 cells (*n* = 3). c) Grayscale analysis quantification of immunoprecipitated M1 protein levels in WT and ADD1-KO PK-15 cells (*n* = 3). d) HEK293T cells were transfected with Flag-ADD1, Flag-ADD1-Δtail, or vector (Flag). At 24 hours post-transfection, p-MLC expression was analyzed by immunoblotting, with grayscale analysis for quantification (*n* = 3). e, f) WT A549 cells were transfected with vector (HA), and ADD1-KO A549 cells were transfected with HA-ADD1, HA-ADD1-Δtail, or vector (HA). At 24 hours post-transfection, cells were stained with phalloidin (green) to label F-actin, immunostained with antibodies against myosin IIB (red) and HA tag (purple), and subjected to fluorescence imaging (e). Phalloidin + myosin IIB: merged images of phalloidin and myosin IIB signals. Merge: merged images of phalloidin, myosin IIB, and HA signals. The Pearson’s correlation coefficient (PCC) of myosin IIB and F-actin was quantified (f) (*n* = 20 cells). Scale bars represent 10 μm. Data are presented as mean ± SD. Statistical analysis was performed using unpaired, two-tailed Student’s t-test. **P*< 0.05; ***P* < 0.01; ****P* < 0.001; *****P* < 0.0001; *ns*, not significant.

**Description of Additional Supplementary Files**

**File Name: Movie S1**

**Description:** PK-15 GFP-RAB5A cells were infected with or without QDs-labeled viruses (red) (MOI = 50) and imaged at 1 hpi. The cells were monitored over a period of 5 min (61 frames) with a time interval of 5 sec. The sample dataset showed fluorescent viruses beneath the plasma membrane appearing to vibrate in place rather than transporting towards the nucleus via RAB5-positive endosomes after ADD1 knockout.

**File Name: Movie S2**

**Description:** PK-15 GFP-RAB7A cells were infected with or without QDs-labeled viruses (red) (MOI = 50) and imaged at 1.5 hpi. The cells were monitored over a period of 5 min (61 frames) with a time interval of 5 sec. The sample dataset showed fluorescent viruses beneath the plasma membrane appearing to vibrate in place rather than transporting towards the nucleus via RAB7-positive endosomes after ADD1 knockout.

**File Name: Movie S3**

**Description:** PK-15 cells were labeled with DiOC18 (green) and infected with or without QDs-labeled viruses (red). The cells were monitored over a period of 2.5 h (51 frames) with a time interval of 3 min. The sample dataset showed that the majority of virus particles resided beneath the plasma membrane, and the trafficking of fluorescent viruses from the plasma membrane to the nucleus was inhibited after ADD1 knockout.

**File Name: Movie S4**

**Description:** PK-15 GFP-RAB5A cells were imaged and the cells were monitored over a period of 5 min (61 frames) with a time interval of 5 sec. The sample dataset showed that RAB5-positive endosomes exhibited random movement at the plasma membrane rather than movement for fusion after ADD1 knockout.

**File Name: Movie S5**

**Description:** PK-15 GFP-RAB7A cells were imaged and the cells were monitored over a period of 5 min (61 frames) with a time interval of 5 sec. The sample dataset showed that the movement of RAB7-positive endosomes exhibited no difference between WT and ADD1-KO cells.

**File Name: Movie S6**

**Description:** The 3D images showed F-actin (green) and viral NP (red) in WT and ADD1-KO cells. Compared to WT cells, more influenza viruses accumulated in actin-rich regions in ADD1-KO cells.

**File Name: Movie S7**

**Description:** PK-15 GFP-lifeact cells were infected with or without QDs-labeled viruses (red) (MOI = 50) and imaged at 1 hpi. The cells were monitored over a period of 5 min (61 frames) with a time interval of 5 sec. The sample dataset showed that fluorescent viruses could break free from F-actin for rapid movement in WT cells, whereas in ADD1-KO cells, the fluorescent viruses appeared to be trapped and unable to move away from F-actin.

**File Name: Movie S8**

**Description:** PK-15 cells were stained with TubGreen™ (green) to label tubulin and infected with QDs-labeled viruses (red) (MOI = 50) and imaged at 1.5 hpi. The cells were monitored over a period of 75 sec (15 frames) with a time interval of 5 sec. The sample dataset showed that the movement of viruses on microtubule exhibited no difference between WT and ADD1-KO cells.

**File Name: Movie S9**

**Description:** PK-15 GFP-lifeact cells were imaged and monitored over a period of 2.5 h (51 frames) with a time interval of 3 min. The sample dataset showed that F-actin dynamics in ADD1 knockout cells seemed to be inhibited.

**File Name: Movie S10**

**Description:** PK-15 GFP-lifeact cells were infected with R18-labeled viruses (red) (MOI = 50) and imaged. The cells were monitored over a period of 2.5 h (51 frames) with a time interval of 3 min. The sample dataset showed that F-actin dynamics in ADD1 knockout cells seemed to be inhibited.

**File Name: Movie S11**

**Description:** A549 GFP-myosin IIB cells were monitored before and after fluorescence photobleaching over a period of 323 s (21 frames), with a time interval of 17 sec between frames after photobleaching. The sample dataset showed that ADD1 knockout inhibited the fluorescence recovery of myosin IIB.

**File Name: Table S1**

RT-qPCR primers, sgRNA primers, cloning primers
